# Upregulated astrocyte HDAC7 induces Alzheimer-like tau pathologies via deacetylating transcription factor-EB and inhibiting lysosome biogenesis

**DOI:** 10.1186/s13024-025-00796-2

**Published:** 2025-01-13

**Authors:** Jinwang Ye, Suyue Zhong, Huali Wan, Xing Guo, Xuanbao Yao, Qiong Liu, Liming Chen, Jian-Zhi Wang, Shifeng Xiao

**Affiliations:** 1 College of Life Sciences and Oceanography, Brain Disease and Big Data Research Institute, Shenzhen University, Shenzhen, 518060 Guangdong China; 2 Department of Laboratory Medicine, Guangdong Provincial People’s Hospital (Guangdong Academy of Medical Sciences), Southern Medical University, Guangzhou, 510000 Guangdong China; 3 Shenzhen-Hong Kong Institute of Brain Science-Shenzhen Fundamental Research Institutions, Shenzhen, 518055 China; 4 Clinical Research Center for Neurological diseases, Shenzhen University, Shenzhen, 518060 Guangdong China; 5 Department of Pathophysiology, School of Basic Medicine, Key Laboratory of Education Ministry of China/Hubei Province for Neurological Disorders, Tongji Medical College, Huazhong University of Science and Technology, Wuhan, 430030 China

**Keywords:** HDAC7, TFEB, Astrocytes, Lysosomal biogenesis, Tau pathology

## Abstract

**Background:**

Astrocytes, the most abundant glial cell type in the brain, will convert into the reactive state in response to proteotoxic stress such as tau accumulation, a characteristic feature of Alzheimer's disease (AD) and other tauopathies. The formation of reactive astrocytes is partially attributed to the disruption of autophagy lysosomal signaling, and inhibiting of some histone deacetylases (HDACs) has been demonstrated to reduce the molecular and functional characteristics of reactive astrocytes. However, the precise role of autophagy lysosomal signaling in astrocytes that regulates tau pathology remains unclear.

**Methods:**

We investigated the expression of class IIa HDAC7 in astrocytes from AD patients and PS19 mice. PS19 mice were treated with AAVs expressing shRNA for HDAC7 with astrocyte-specific promoter and with a selective class IIa HDAC inhibitor, TMP195, and the effects on tau pathology, gliosis, synaptic plasticity and cognition-related behavioral performance were measured. Tau uptake and degradation assays in cultured astrocytes were utilized to investigate the role of HDAC7 on astrocyte-mediated tau clearance. Immunoprecipitation, immunofluorescence, western blotting, RT-qPCR, mass spectrometric, and luciferase reporter assay were used to identify HDAC7 substrates, modification site and related signaling pathways in astrocyte-tau clearance. We generated a new antibody to clarify the role of HDAC7-mediated signaling in AD patients and PS19 mice.

**Results:**

Here, we found that the level of histone deacetylase 7 (HDAC7) was remarkably increased in the astrocytes of AD patients and P301S tau transgenic (PS19) mice. Genetic or pharmacological inhibition of HDAC7 effectively enhanced astrocytic clearance of tau with improved cognitive functions in PS19 mice. HDAC7 could modulate astrocytic uptake and lysosomal degradation of tau proteins through a transcriptional factor EB (TFEB) acetylation-dependent manner. Specifically, deacetylation of TFEB at K310 site by HDAC7 prevented TFEB nuclear translocation with reduced lysosomal biogenesis and tau clearance in astrocytes, whereas inhibiting HDAC7 restored astrocytic TFEB acetylation level at K310 with improved tau pathology and cognitive functions in PS19 mice.

**Conclusions:**

Our findings suggest that upregulation of HDAC7 induces AD-like tau pathologies via deacetylating TFEB and inhibiting lysosomal biogenesis in astrocytes, and downregulating HDAC7-TFEB signaling is promising for arresting AD and other tauopathies.

**Supplementary Information:**

The online version contains supplementary material available at 10.1186/s13024-025-00796-2.

## Background

Tauopathies are a class of neurodegenerative diseases characterized by accumulation of neurofibrillary tangles (NFTs) composed of hyperphosphorylated and misfolded tau protein [1]. In Alzheimer’s disease (AD), the most common form of tauopathy, the level of NFTs burden is strongly correlated with neuronal loss, neurodegeneration and progressive cognitive decline [2, 3]. Intraneuronal tau aggregates can be released to the extracellular space and captured by recipient cells, resulting in prion-like propagation of tau pathology [4]. Cumulative evidence has shown that astrocytes participate in tau pathology and propagation, but their exact roles remain unclear. In the brain of AD patients and tauopathy models, astrocytes undergo morphological and molecular changes and switch to a “reactive” state, which may dynamically change the balance between their neuroprotective and neurotoxic properties [5]. It has been suggested that reactive astrocytes might protect against tau pathology as they can directly uptake extracellular tau and facilitate tau clearance in virtue of their close proximity to NFTs [6, 7]. Astrocytes can uptake and degrade tau preformed fibrils (tau-pff) in vitro [7]. Spreading of tau from neurons to astrocytes also has been observed in mouse models [8]. Analysis of postmortem tauopathy brains have consistently observed the accumulation of tau-containing granules in astrocytes [9, 10], and increased presence of tau in astrocytes is associated with tau immunotherapy in progressive supranuclear palsy (PSP) patients [11].

Impairment of autophagy-lysosomal pathway has been linked to astrocytic clearance dysfunction in neurodegenerative diseases. Loss of astrocytic autophagy exacerbates amyloid-β (Aβ) plaque formation and cognitive impairments in APP/PS1 mouse model [12]. ApoE4 astrocytes from human iPSCs exhibit impaired autophagy-lysosomal activity and cholesterol accumulation [13]. Transcriptional factor EB (TFEB) is a master regulator of lysosomal biogenesis and autophagy [14], which regulates the transcription of autophagic and lysosomal genes by directly binding to the coordinated lysosomal expression and regulation (CLEAR) motifs [14, 15]. TFEB plays critical role in the uptake and degradation of Aβ oligomers and tau fibrils in cultured astrocytes [7, 16]. Astrocyte-specific overexpression of TFEB hinders the buildup and spreading of tau pathology by bolstering lysosomal elimination within astrocytes in a tauopathy mouse model [7]. Activating astrocytic TFEB has also been demonstrated to reduce Aβ burden in the 5xFAD mouse model [17]. However, the precise mechanisms underlying the impairment of astrocytic autophagy-lysosomal signaling in tau pathology remain unclear.

Class IIa histone deacetylases (HDACs) are potential therapeutic targets in neurodegenerative diseases. Class IIa HDACs (HDAC4, 5, 7 and 9) shuttle between the cytoplasm and nucleus to regulate neuronal survival, synaptic plasticity and memory formation [18, 19]. HDAC4 interacts with mutant huntingtin and colocalizes with cytoplasmic inclusions in Huntington's disease [20]. Nuclear accumulation of HDAC4 promotes neurodegeneration in Ataxia telangiectasia and Parkinson’s disease [21, 22]. Inhibition of class IIa HDACs protects against neurodegeneration and cell death in Parkinson’s disease model [23]. Moreover, HDAC4 inhibition attenuates Aβ clearance deficits in ApoE4 astrocytes by epigenetic correction of endosomal pH [24]. Particular in astrocytes, we have previously reported that HDAC7 is involved in LPS-induced astrocyte activation and inflammatory responses [25]. However, the relation between Class IIa HDACs and tau pathology hasn’t been yet reported to our knowledge.

In the current study, we found that the expression of HDAC7 is increased specifically among the four class IIa HDACs in the astrocytes of PS19 mice and AD patients. Genetic knockdown or pharmacological inhibition of HDAC7 ameliorated tau pathology, synaptic impairments and cognitive deficits in PS19 mice. Knockout/inhibition of HDAC7 in cultured astrocytes enhanced tau fibril uptake and degradation and lysosomal activity. Mechanically, we identified that HDAC7 directly binds to and deacetylates TFEB at a newly-discovered K310 site, resulting in cytoplasmic TFEB retention and impaired lysosomal biogenesis. We further showed that acetylation at K310 in TFEB is a potential driver event in mediating astrocyte-mediated tau clearance. Together, our study suggests that HDAC7 in astrocytes regulates tau clearance via the control of TFEB lysosomal pathway, and inhibition of astrocytic HDAC7 is a potential therapeutic strategy for AD and other tau-mediated neurodegeneration.

## Methods

### Viruses, reagents and antibodies

Human TFEB was subcloned into pCDNA3.1-CMV-MCS-3flag plasmid, and single, double or triple point mutation based on the above plasmid were constructed by SyngenTech Biotechnology (Beijing, China). The TFEB luciferase plasmid was constructed by Obio Technology (Shanghai, China). The HDAC7 and P301S-tau (1N4R) plasmids, and the Lenti-GfaABC1D-Cre-NLS, LV-GfaABC1D-HDAC7-GFP, LV-GfaABC1D-GFP, LV-GfaABC1D-TFEB (WT)−3xFlag, LV-GfaABC1D-TFEB (S122D)−3xFlag, LV-GfaABC1D-TFEB (S211D)−3xFlag, LV-GfaABC1D-TFEB (K310Q)−3xFlag, LV-GfaABC1D-TFEB (K310R)−3xFlag, LV-GfaABC1D-3xFlag, LV-GfaABC1D-Tau (human P301S, 1N4R)−3xFlag and LV-GfaABC1D-mCherry-Tau (human P301S, 1N4R) were constructed by Obio Technology (Shanghai, China). The AAV-G1-shHD7-GFP and LV-GfaABC1D-GFP were constructed and packaged by Brain Case Technology (Shenzhen, China), and the target sequence of HDAC7 shRNA is TGCGCTACAAACCCAAGAAAT. The AAV11 was used in animal experiments. Lentiviruses were used in primary astrocytes with Multiplicity of infection (MOI) of 10. TMP195 (a selective class IIa HDAC inhibitor), Bafilomycin A1 (a H^+^-ATPase inhibitor) and Torin1 (a mTORC1 inhibitor) were bought from Selleck (Houston, TX, USA). LysoTracker Red DND-99 was from Invitrogen (Carlsbad, CA, USA). Antibodies used in this study are listed in Supplementary Table 1.

### Human samples

Dr. Chao Ma from the Human Brain Bank at the Chinese Academy of Medical Sciences and Peking Union Medical College (Beijing, China) kindly provided postmortem human brain samples. The diagnosis of Alzheimer's disease (AD) was conducted in accordance with the criteria established by the consortium to Establish a Registry for AD and the National Institute on Aging. This study was performed in accordance with institutional regulatory guidelines and principles of human subject protection in the Declaration of Helsinki. Detailed information regarding the human samples can be found in Supplementary Table 2.

### Animals, stereotaxic surgery and drug treatment

The PS19 mice (PS19 line, Stock No: 008169) were obtained from The Jackson Laboratory. Heterozygotes were bred to C57BL/6 wild-type mice to maintain the line. HDAC7^flx/flx^ mice were obtained from GenPharmatech. Adult C57BL/6 mice were obtained from Changzhou Cavens Laboratory Animal Co., Ltd (Changzhou, China). The mice were housed in groups of four to five per cage with ad libitum access to food and water, and were maintained under a 12-h light/dark cycle (lights on at 7:00 p.m., off at 7:00 a.m.) at a stable temperature (22 ± 2 °C). In the present study, we have complied with all relevant ethical regulations for the animal testing and research. All procedures were approved by institutional guidelines and the Animal Care and Use Committee (Shenzhen University, Shenzhen, China) of the university’s animal core facility.

For brain stereotactic injection, mice were anesthetized with isoflurane and placed on a stereotaxic apparatus, and then sterilized with iodophor and the scalp was incised along the midline of the head. Hole was stereotaxically drilled in the skull at posterior 2.1 mm, lateral 1.4 mm, and ventral 2.0 mm relative to the bregma. Using a microinjection system (World Precision Instruments), LV-GfaABC1D-HDAC7-GFP (2.5 μL, 1.0 × 10^9^ vg/mL), LV-GfaABC1D -GFP (2.5 μL, 1.0 × 10^9^ vg/mL), AAV-GfaABC1D-shHDAC7-GFP (1μL, 5.0 × 10^12^ vg/mL) or AAV-GfaABC1D-shHDAC7-GFP (1μL, 5.0 × 10^12^ vg/mL) was bilaterally injected into the hippocampal DG region (posterior 2.1 mm, lateral 1.4 mm, ventral 2.0 mm) at a rate of 0.125 μL/min. The needle was kept for 5 min before withdrawal, the skin was sutured and mice were placed beside a heater for recovery.

For intraperitoneal injection, TMP195 was diluted to 7.5 mg/mL with sterile 0.9% saline containing 5% (vol/vol) Tween-80 and 20% (vol/vol) PEG-300. TauTg or WT mice were then intraperitoneally injected with the diluted TMP195.

### Novel object recognition

Arenas (50cm × 50cm × 50 cm) were utilized to house object A and object B in the corner. For the training trials, the mice were given a 5-minute period to acclimate themselves on the first day. Following each familiarization period, the arenas were cleansed with 70% ethanol. After a 24-hour interval, object B was substituted with object C, and the mice were allotted 5 minutes to investigate both objects. The behavioral responses were documented via a video camera situated overhead the arenas, and the duration of exploration in the arena containing the novel object C and familiar object A was quantified respectively.

### Morris water maze

The Morris Water Maze (MWM) test was employed to assess spatial learning and memory, as previously described [26]. During the acquisition training phase, mice underwent daily training in the water maze for a duration of 5 consecutive days, with each day consisting of 3 trials. These trials were conducted between 14:00 and 20:00 p.m., with a 30-second interval between each trial. In each trial, mice were allowed 60 s to find the hidden platform. In the event that the mice were unable to locate the platform within 60 s, they were guided to it and required to remain on it for 30 s. The time taken to find the platform and the swimming path within the 60 s timeframe were recorded using a fixed video camera positioned 1.5 meters above the water surface. Spatial memory was assessed 48 h after the training phase, wherein the platform was removed. A digital device connected to a computer was employed to record the latency of the initial platform crossing, the number of target platform crossings within the 60-second period, and the time spent in the target quadrant during the 60-s probe phase.

### Electrophysiology recordings

Mice were anesthetized with isoflurane and brains were removed in ice-cold artificial cerebrospinal fluid (a-CSF): 119 mM NaCl, 2.5 mM KCl, 26.2 mM NaHCO_3_, 1 mM NaH_2_PO_4_, 11 mM glucose, 1.3 mM MgSO_4_, and 2.5 mM CaCl_2_ (pH 7.4). Coronal slices (350 μm thick) were cut in ice-cold a-CSF using a Leica VT1000S vibratome and then transferred into an oxygenated chamber for a 2 h recover at room temperature.

For LTP recordings, brain slices were transferred to a recording chamber and immersed in a-CSF. The slices were placed in a chamber containing an 8 × 8 microelectrode array (Parker Technology, Beijing, China) on the bottom surface, with each microelectrode measuring 50 × 50 um and spaced 150 μm apart. The slices remained submerged in a-CSF throughout the experiment. Electrical signals were captured using the MED64 system (alpha MED Sciences, Japan). Stimulation of the Schaffer collaterals from the CA3 region was achieved using a 0.1MΩ tungsten monopolar electrode, while field excitatory postsynaptic potentials (fEPSPs) in the CA1 region were recorded using a glass microelectrode filled with a-CSF with a resistance of 3–4 MΩ. The signals were amplified utilizing a MultiClamp 700 B amplifier (Axon), digitized through a Digidata 1440A (Axon) with a 2 kHz low pass filter and a 3 Hz high pass filter, and subsequently recorded and saved using Clampex 10.4 software (Axon) for subsequent offline data analysis. LTP of fEPSPs was induced by three times of high-frequency stimulation (HFS; 100Hz, 1-s duration) with a 200-ms interval. The LTP magnitude was assessed as the normalized percentage change in the fEPSP slope (10%−90%) taken during the 60 min interval after LTP induction.

### Golgi staining and analysis

Golgi staining was performed according to the manufacturer's protocol (FD NeuroTech, cat# PK401). Briefly, mouse brains were removed after anesthetization with isoflurane and immersed in a mixture of Solutions A + B for 2 weeks at room temperature in dark. Then the brains were transferred into a new tube and added with Solution C for another 7 days at 4°C in dark. All brains were sliced into 100‐μm‐thick sections using a Vibratome (VT1200S; Leica, Germany). Images were obtained using a microscope (Ni‐E, Nikon, Japan). The dendritic complexity of hippocampal DG neurons was assessed through Sholl analysis utilizing the Simple Neurite Tracer plugin and ImageJ (Fiji, Japan) software. Imaging of dendritic spines of DG neurons was conducted using a 100× oil immersion lens. The analysis in this study focused solely on secondary dendrites.

### Cell culture

Primary mouse astrocytes were isolated and purified from the mouse cortex as described previously [26]. In brief, cortices from C57BL/6 or HDAC7^flx/flx^ mice were isolated and dissociated cells were centrifuged and resuspended in Dulbecco’s modified Eagle’s medium (DMEM) F-12 supplemented with 10% Fetal bovine serum (FBS) and 1% penicillin/streptomycin (complete medium). The cells were then plated in a T75 flask, and the culture medium was changed every 3 days. 7–10 days later, the astrocytes were sorted at shake cultivation rotating for 14 h at 200–220 rpm. The cultured cells were then treated with trypsin and the disassociated cells were re-plated in culture plate or 12-well glass chambers at a density of 5 × 10^5^ cells per well for Western blotting and quantitative real-time PCR (RT-qPCR) analysis and 1 × 10^5^ cells per well for immunofluorescence imaging.

HEK293T cells were cultured with DMEM-high glucose medium supplemented with 10% Fetal FBS and 1% penicillin/streptomycin at 37℃ in the presence of 5% CO_2_. HEK293T cell transfection was performed with lipoFactMaxTM (ABP biosciences, Beltsville, USA).

### Western blotting

Western blotting was performed as previously described [25]. To analyze soluble and insoluble tau fractions in mice, mouse brain tissues were extracted following an established protocol [27]. Briefly, mouse brain tissues were isolated on ice-cold PBS, homogenized in RIPA lysis buffer (Beyotime, cat# P0013B) with 1% protease inhibitors and phosphatase inhibitors, and centrifuged at 14,000 g for 15 min at 4°C. Then, the supernatant was transferred to new tubes. The pellet was washed with ice-cold RIPA buffer and then lysed with 10% SDS buffer (10% SDS, 250 mM Tris, pH 6.8) and sonicated in a water bath sonicator until no particulate material was visible. Protein concentration was measured using the BCA method (Pierce BCA protein assay kit, 23225) for both RIPA-soluble and RIPA-insoluble extracts. After SDS/PAGE, the protein was transferred onto nitrocellulose membranes (Whatman) and then blocked with blocking buffer (Beyotime, cat# P0023B) for 1 h at room temperature. Indicated primary antibodies were incubated at 4℃ overnight. The corresponding peroxidase-conjugated secondary antibodies were incubated for 1 h at room temperature. Protein signals were detected by the ECL detection system and analyzed with ImageJ software.

### Immunoprecipitation

The immunoprecipitation assay was conducted in accordance with established protocols. Briefly, cultured cells were lysed on ice for 30 minutes using IP buffer (Beyotime, cat# P0013) containing 1% protease inhibitors and phosphatase inhibitors, followed by centrifugation at 14,000 g for 10 minutes. The resulting supernatants (1 μg protein) were then incubated overnight at 4°C with specified primary antibodies, with gentle rocking, and subsequently incubated for 2 hours with protein A + G agarose. The immunoprecipitates were washed three times with PBS, resuspended in 2× loading buffer, eluted by boiling for 10 minutes and analyzed by western blotting.

### Immunostaining

For animal experiments, mice were subjected to anesthesia using a 1% solution of pentobarbital sodium, followed by intracardial perfusion with saline and subsequently with a 4% solution of paraformaldehyde (PFA) in 0.1 M phosphate buffer at a pH of 7.4. The brains of the mice were then extracted and further fixed in a 4% PFA solution for a duration of 12 hours, after which they were cryoprotected using successive solutions of 20% and 30% sucrose. Brain sections with a thickness of 35 μm were obtained using a cryostat microtome (CM1900, Leica, Germany). In order to perform immunofluorescence staining, the aforementioned brain sections were first washed in PBS, then blocked in a buffer solution containing 3% bull serum albumin and 0.5% Triton X-100 for a period of 1 hour, and subsequently incubated with primary antibodies at 4℃ overnight. After washed with PBS, slices were incubated with secondary antibodies conjugated to Alexa-Fluor 488/546/647 at 37℃ for 1 h, followed by DAPI staining for 10 min.

For immunohistochemistry staining, endogenous peroxidase activity was eliminated by incubating brain slices in 0.3% H_2_O_2_ (in PBS) at 37℃ for 30 min before serum blocking. Immunoreactions were performed using a DAB-staining kit (ZSGB-BIO). For immunostaining of human brain slices, antigen-retrieval was performed in boiling sodium citrate buffer (10 mM, pH 6.0) and auto fluorescence of the brain slices was blocked with Sudan black (0.3%, room temperature, 10 min). For primary astrocytes, after washed with PBS, cells were fixed with paraformaldehyde (4%, room temperature, 20 min) and permeabilized in PBS containing triton X-100 (0.5%, 30 min) before serum blocking.

The slices or coverslips were washed with PBS and mounted onto slides. Imaging was performed using a Zeiss LSM880 confocal microscope or Leica Aperio CS2 scanner.

### Mass spectrometric analysis

The mass spectrometric (MS) analysis was performed based on previous report [28]. Briefly, Flag-TFEB together with HDAC7 or vector was transfected into HEK293T cells for 24 h, and then immunoprecipitation was conducted with Flag antibody as described above. The immunoprecipitated protein samples were separated by SDS-PAGE and stained by Coomassie brilliant blue staining, and then the interested bands were carefully cut off and digested with trypsin. The peptides were analyzed with a ThermoFisher Q-Exactive mass spectrometer (ThermoFisher, USA) fitted with a Nano Flex ion source (Thermo Fisher, USA). The LC-MS/MS data were subjected to analysis for the purpose of protein identification and quantification utilizing PEAKS Studio 8.5. The local false discovery rate at PSM was 1.0% after searching against the Uniprot human database with a maximum of two missed cleavages. Precursor and fragment mass tolerance were set to 10 ppm and 0.05 Da, respectively. Shortlisted acetylated peptides were further subjected to manual examination of MS/MS spectra to identify the precise locations of acetylation.

To assess the distribution of TMP195 in the brain after intraperitoneal administration, the mouse brain was harvested at various time points (2, 4, 6, 8, 10 and 12 h post-injection) and immediately immersed in methanol. Subsequently, the brain tissue was homogenized at 4°C, followed by centrifugation at 14,000 g for 15 min and centrifugation at 30,000 g for 30 min. The supernatant along with a TMP195 standard, was subjected to LC-MS/MS analysis using AB SCIEX QTRAP6500+. The content of TMP195 in each sample was determined by referencing a standard curve.

### Tau fibril preparation

The untagged full-length 2N4R human tau was cloned into the pET29b vector in the *E. coli* strain BL21. Briefly, BL-21 cells transfected with tau were cultured for 12–16 h in LB medium containing 100 μg/mL ampicillin at 180 rpm, at 37 °C. The cells were then collected and lysed by sonication (5 s sonication, 2 s interval, total 15 min) in bacterial lysate with 1 mM PMSF. Sequentially, the tau protein was purified by anion exchange chromatography (HiPrep CM FF 16/10, Uppsala, Sweden) and agarose chromatography (Hiload 16/600 Superdex 75 pg, Uppsala, Sweden). The buffer used for tau purification is as following: for anion exchange chromatography, start buffer: 25 mM Tris-HCl, 20 mM NaCl, pH 8; elution buffer: 25 mM Tris-HCl, 1 M NaCl, pH 8. For agarose chromatography, running buffer: 0.05 M NaPO4, 0.15 M NaCl, pH 7.2. A fast protein liquid chromatography system (AKTA, GE Healthcare, Boston, MA, USA) was used in this study. Coomassie brilliant blue staining was used to identify the purified fractions. The in vitro tau fibrillization assay was performed under the following conditions: 50 μM purified tau protein, 12.5 μM heparin, 2 mM dithiothreitol (DTT), and a protease inhibitor cocktail comprising 10 μg/ml leupeptin, 5 μg/ml chymostatin, 3 μg/ml elastatinal, and 1 μg/ml pepstatin, all in phosphate buffer saline (PBS). The mixed solution was incubated at 37℃ for a duration of two weeks, with the addition of 1 mM fresh DTT to the solution every 24 h. Fibrillization was confirmed using the thioflavin T fluorescence assay, Coomassie brilliant blue staining and transmission electron microscopy. For transmission electron microscopy, the protein samples were diluted to 5 μM and spotted onto a 230-mesh carbon-coated copper grid. At 10,000 x magnification, pffs were imaged using a Jeol JEM1230 transmission electron microscope. After incubation for 30 min, the residual solution on the surface of the grid was removed by filter paper. The grids were washed with ddH_2_O and mixed with 1% uranium acetate for 30 s. After confirmation, the pffs were frozen as single use aliquots (50 μM) at −80℃. The purified tau fibrils were labelled with 5-FAM label at 4℃ for 8 h, and dialysis for 72 hours (dialysate was changed three times) to remove unbound 5-FAM. The specific absorption peak at 490 nm of FAM-labeled tau fibrils was further confirmed.

### Tau uptake and degradation assay

Primary astrocytes were incubated with FAM-labelled tau-pff (1 μM) for 4 h, then washed with PBS three times, followed by incubation of lysotracker red for 30 min (100 nM) and staining with Hoechst for 5 min. Cells were subjected to live imaging using a Zeiss LSM880 confocal microscopy. The colocalization of tau-pff and Lysotracker was quantified with ImageJ software using Manders' Colocalization Coefficients. the M_1_ Manders' coefficient is defined as: ${M}_{1}=\frac{{\Sigma }_{i}{C1}_{i,Colocal}}{{{\Sigma }_{i}C1}_{i}}$, where ${C1}_{i}$ represent the intensity of individual pixels for tau-pff, and ${C1}_{i,Colocal}$ represent the colocalizing pixels of tau-pff with Lysotracker. For tau uptake assay, astrocytes were incubated with tau-pff (1 μM) for 1–12 h. At various time points, cells were washed with PBS three times and lysed in RIPA buffer. Intracellular tau levels were quantified by ELISA analysis.

For tau degradation assay, astrocytes were incubated with tau-pff (1 μM) for 4 h, followed by the removal of the tau-containing media and thorough washout. Cells were continuously cultured at 37℃ for 30–180 min. At various time points, cells were washed with PBS three times and lysed in RIPA buffer. Intracellular tau levels were quantified by ELISA analysis. Bafilomycin A1 (100 nM) was added to the cells 1 h before washout of tau-containing media and cells were cultured in its presence for 30–120 min until ELISA analysis.

### ELISA

The levels of intracellular tau in primary mouse astrocytes following incubation with tau-pff were analyzed with a total tau ELISA kit (57519, Cell Signaling Technology, Danvers, MA, USA). Experiment was performed according to the manufacturer’s protocol.

### Quantitative real-time PCR (RT-qPCR)

The total RNA of astrocytes was extracted using the TRIzol (Invitrogen, cat# 15596026) and reverse transcription was conducted using the PrimeScript real time Master Mix (TAKARA, cat# RR047A). Total reaction volume of RT-qPCR systems is 20 μl containing 10 μL 2◊ SYBR Green Master Mix (AG11701, AGbio), 2 μL forward primer (2 mM), 2 μL reverse primer (2 mM), 5 μL RNase/DNase-free H_2_O, and 1 μL pre-amplified cDNA (100 ng/μl). Samples were assayed in a BIO-RAD CFX96 RT-qPCR system. Primer sequences used for RT-qPCR can be found in Supplementary Table 3.

### Luciferase reporter assay

The HDAC7 (or vector) or shHD7 (or scramble), TFEB-3xFlag, pTFEB‐Luc reporter and Renilla luciferase (pRL‐TK) constructs were transfected into HEK293T cells by LipoFectMax. 24 h after transfection, the cells were harvested and lysed in 100 μL Passive Lysis Buffer. Luciferase activity was analyzed using a Lumat LB9507 luminometer (Berthold, Germany) and the Dual-luciferase Reporter Assay Systerm (Promega, Madison, WI, USA) according to the manufacturer’s protocol. Relative light units of TFEB luciferase were normalized to Renilla luciferase light units to control for transfection efficiency.

### Statistical analysis

Statistical analyses were performed with Graphpad Prism 9 (LaJolla, CA, USA). Unpaired Student’s t-test was used to determine differences between two groups. One-way or two-way analysis of variance (ANOVA) followed by Tukey’s multiple comparisons test was used to determine differences among three or more groups as indicated in the figure legends. The statistically significance levels were set at *p* < 0.05 (*), *p* < 0.01 (**), *p* < 0.001 (***) with a confidence interval of 95%. Data were expressed as mean ± SEM. All samples or animals were included for statistical analysis unless otherwise noted in pre-established criteria.

## Results

### HDAC7 is increased in astrocytes of the AD patients and PS19 mice

**Fig. 1:**
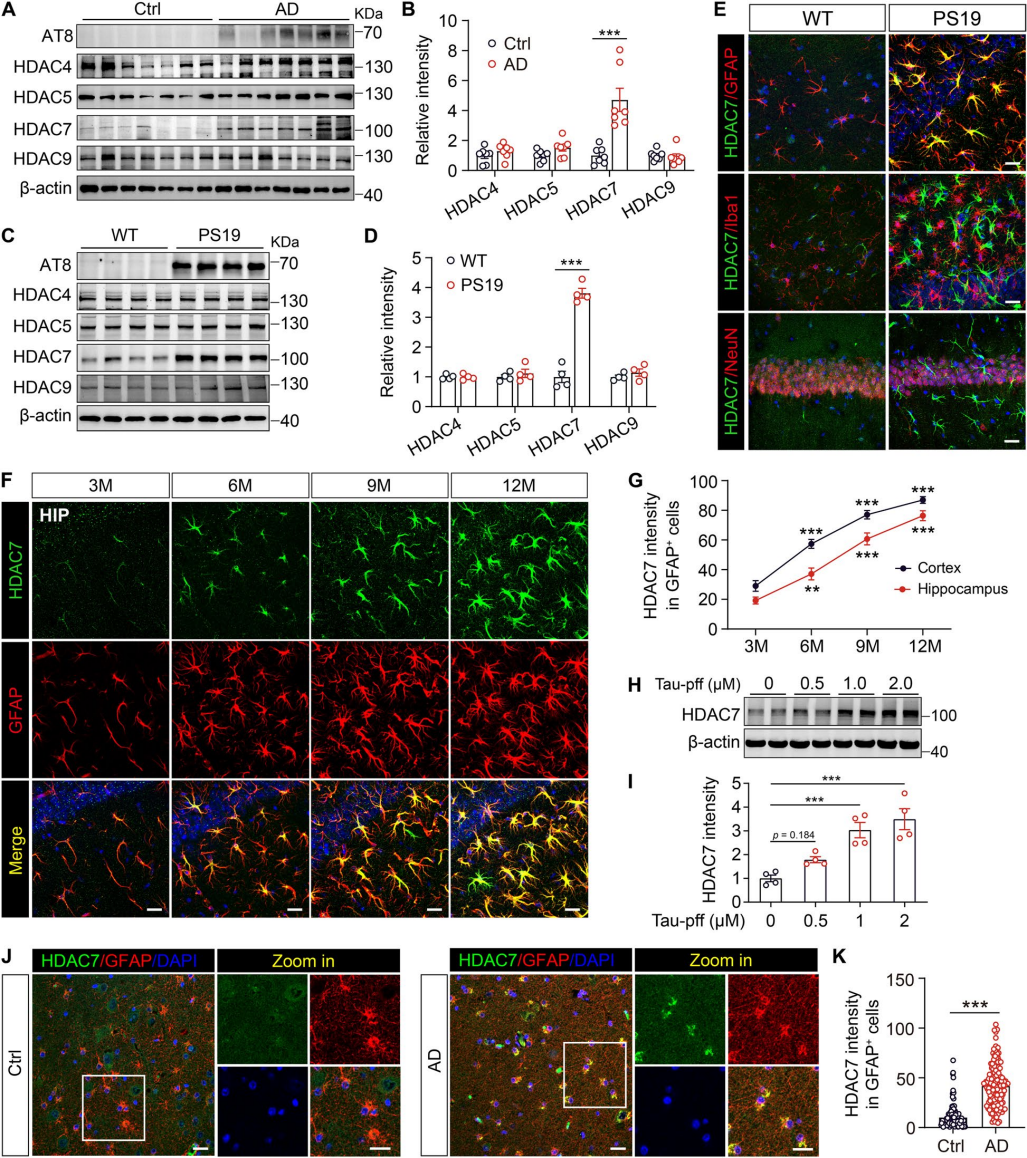
HDAC7 level is increased in astrocytes of AD patients and PS19 mice. **A** Western blotting analysis of AT8 and class IIa HDACs (HDAC4, 5, 7 and 9) in the brain lysates of AD patients and control individuals. **B** Quantification of the blots in A. *n* = 7 per group. **C** Western blotting analysis of class IIa HDACs (HDAC4, 5, 7 and 9) in the hippocampal lysates of 8-month-old WT and PS19 mice. **D** Quantification of the blots in C. *n* = 4 per group. **E** Representative immunostaining images of HDAC7 and glial fibrillary acidic protein (GFAP)/ionized calcium binding adapter molecule 1 (Iba1)/neuronal nuclei (NeuN) in the brain of 8-month-old WT and PS19 mice. Scale bar: 20 μm. **F** Representative immunostaining images of HDAC7 and GFAP in the hippocampus of 3, 6, 9 and 12-month-old PS19 mice. Scale bar: 20 μm. **G** Quantifications of HDAC7 intensity in GFAP^+^ cells in the hippocampus and cortex (representative images are shown in Supplementary Fig. 1) of PS19 mice. *n* = 7 sections from 3 mice. **H**, **I** Western blotting analysis and quantification of HDAC7 in primary astrocytes treated with various concentration of tau-pff for 24 h. *n* = 4 per group. **J** Representative immunostaining images of HDAC7 and GFAP in the brain sections of AD patients and control individuals, **K** quantification of HDAC7 intensity in GFAP^+^ cells, *n* = 135 (Ctrl), 160 (AD) cells from 6 sections per group. Scale bar: 20 μm. Statistical significance was determined by unpaired Student’s t test (B, D, G and K) or one-way ANOVA with Tukey’s post hoc analysis (I). Data are shown as mean ± SEM, *p < 0.05, **p < 0.01, ***p < 0.001, n. s, not significant

Our previous study has demonstrated that class IIa HDACs play a critical role in astrocyte activation and inflammation, which is a well-known pathological feature of AD [25]. To investigate the potential role of class IIa HDACs in AD pathology, we firstly analyzed their protein levels in postmortem brain tissues of AD and non-AD individuals. Western blot analysis showed that HDAC7 is selectively increased in the hippocampal lysates of AD patients, with HDAC4, HDAC5 and HDAC9 unchanged (Fig. 1A, B). Analysis of the reprocessed RNA-seq data from the hippocampus (GSE28146, GSE29378, GSE36980, GSE48350, GSE5281) and entorhinal cortex (GSE26927, GSE26972, GSE48350, GSE5281) in the AD dataset also revealed that, HDAC7 gene expression is markedly increased in both the hippocampus and entorhinal cortex of AD patients compared with control (Supplementary Fig. 1A).

To determine whether this phenomenon can be detected in mouse models of tauopathy, we next assessed class IIa HDAC levels in the widely-used PS19 tau transgenic mice, which overexpress P301S tau under the prion promotor and display widespread NFTs, gliosis and cognitive decline by six months of age [29]. HDAC7 mRNA level was significantly upregulated in the hippocampus of both 6- and 9-month-old PS19 mice as compared with age-matched WT mice (Supplementary Fig. 1B). Western blot analysis also showed that the protein level of HDAC7 among class IIa HDACs is selectively upregulated in the hippocampus of 9-month-old PS19 mice (Fig. 1C, D). To identify changes in HDAC7 in different brain cell types, we performed immunofluorescence co-staining of HDAC7 along with antibodies against markers for neurons (NeuN), astrocytes (GFAP), and microglia (Iba1). Immunostaining revealed that elevated HDAC7 mainly colocalize with astrocytes, but not neurons or microglia, in the brain of PS19 mice (Fig. 1E and Supplementary Fig. 1C). Moreover, the expression level of HDAC7 in astrocytes increases in an age-dependent manner in the hippocampus and cortex of PS19 mice (Fig. 1F, G and Supplementary Fig. 1D). By treating with tau-pff, a concentration-dependent upregulation of HDAC7 protein level was also detected in primary astrocytes (Fig. 1H, I). In addition, HDAC7 staining intensity in astrocytes was also significantly increased in brain samples from AD patients compared with samples from healthy individuals (Fig. 1J, K). Taken together, these data indicate that HDAC7 expression increases in reactive astrocytes in AD patients and tau transgenic mice.

### Knockdown of astrocytic HDAC7 reduces tau pathology and attenuates synaptic impairments and cognitive deficits in PS19 mice

**Fig. 2:**
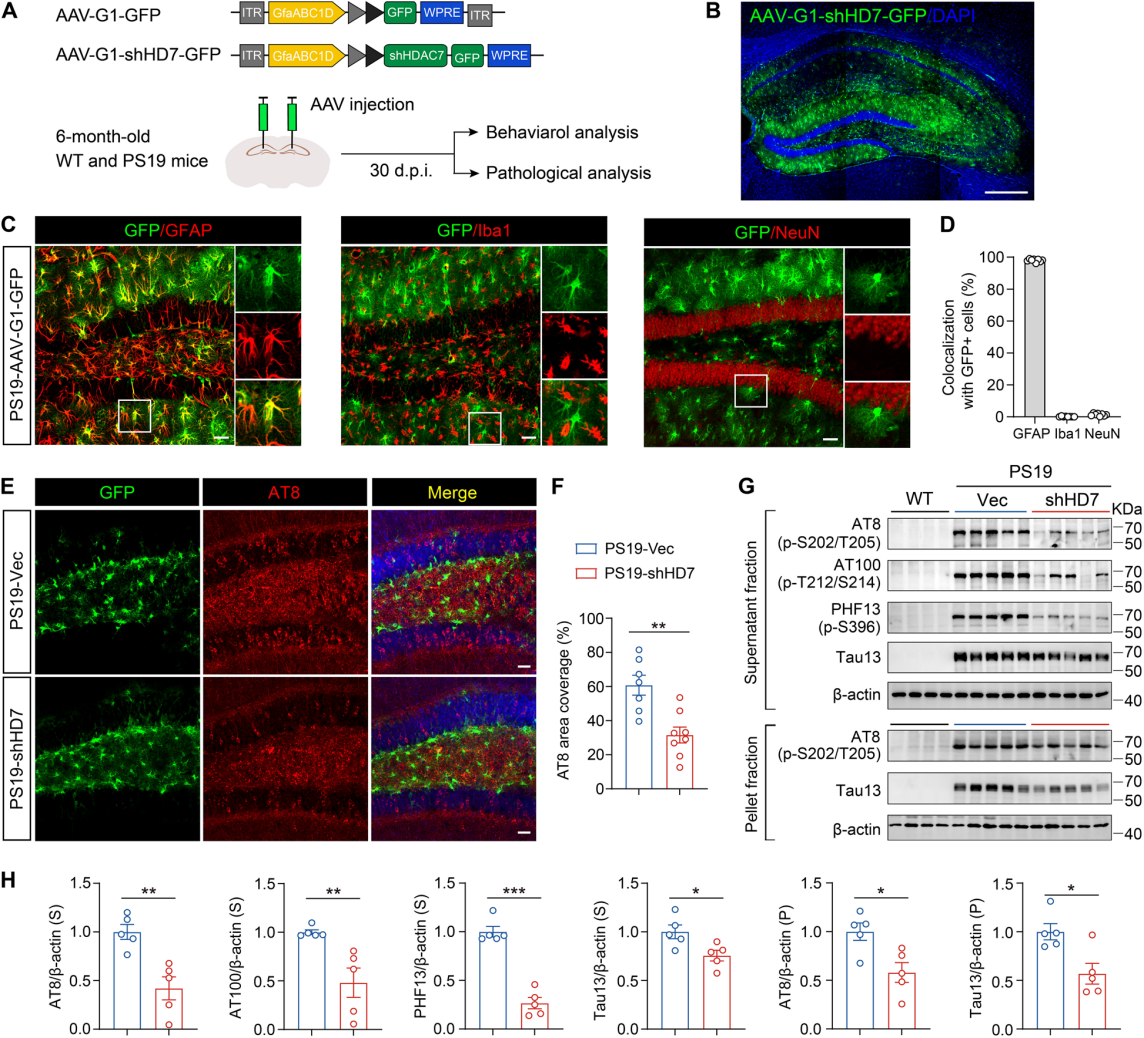
Knockdown of astrocytic HDAC7 reduces tau pathology in PS19 mice. **A** Schematic illustration of AAVs constructed with shRNA targeting the knockdown of HDAC7 or control GFP and the in vivo experimental timeline. **B** Representative images showing the expression of AAV in the hippocampus of PS19 mice. Scale bar: 200 μm. **C**, **D** Representative immunostaining images of GFP and GFAP/ Iba1/NeuN in the hippocampus of 7-month-old PS19 mice, with analysis of the ratio of GFAP^+^ GFP^+^, Iba1^+^ GFP^+^ and NeuN^+^ GFP^+^ double positive cells. *n* = 7 (PS19-Vec), 8 (PS19-shHD7). Scale bar: 40 μm. **E**, **F** Representative immunostaining images with quantification of AT8 in the hippocampus of PS19 mice after AAV injection. *n* = 7 (PS19-Vec), 8 (PS19-shHD7). Scale bar: 40 μm. **G**, **H** Western blotting analysis and quantification of tau accumulation in the supernatant and pellet fraction of hippocampal ex from WT and PS19 mice using AT8, AT100, PHF13 and tau13 antibodies. *n* = 5 per group. Statistical significance was determined by unpaired Student’s t test. Data are shown as mean ± SEM, *p < 0.05, **p < 0.01, ***p < 0.001, n. s, not significant

To explore the role of astrocytic HDAC7 in the progression of tau pathology, an adeno-associated virus (AAV) was engineered to express a small hairpin RNA (shRNA) targeting HDAC7 (shHD7) under the astrocyte-specific promoter GfaABC1D (G1). Then we microinjected AAV-G1-shHD7-GFP or AAV-G1-GFP into the hippocampus of 6-month-old WT and PS19 mice (Fig. 2A), a time point characterized by prominent elevation of HDAC7 level in astrocytes in PS19 mice (Fig. 1F, G). After one month, the expression of the astrocyte-specific AAV in the hippocampus was confirmed by confocal microscopy (Fig. 2B). Immunostaining conducted one-month post injection revealed that almost all GFP signals (97.74%) were co-localized with astrocytes (GFAP) rather than microglia (Iba1) or neurons (NeuN), verifying the specific expression of the AAVs in astrocytes (Fig. 2C, D). Efficacy for the knockdown of HDAC7 with the AAV was confirmed by immunostaining with HDAC7 (Supplementary Fig. 2A, B). Immunostaining analysis using AT8 (phospho-tau202/205) antibody showed that knockdown of astrocytic HDAC7 greatly reduced AT8^+^ tau level in the hippocampus of PS19 mice (Fig. 2E, F). Furthermore, we observed a dramatic decrease in levels of AT8, AT100, PHF13 and tau 13 (total human tau) in the soluble fraction, and levels of AT8 and tau13 in the insoluble fraction in hippocampal extracts from PS19-shHD7 mice compared to PS19-Vec mice (Fig. 2G, H). These results indicated that knockdown of HDAC7 in astrocytes can protect against tau pathology in tau transgenic mice.

**Fig. 3:**
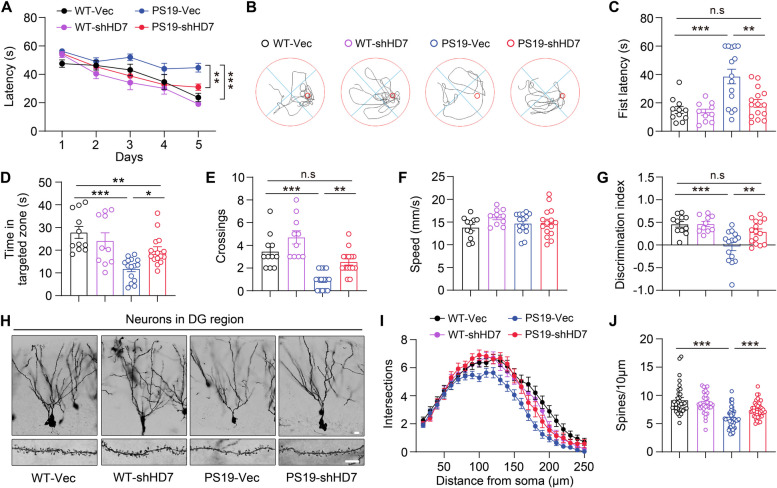
Knockdown of astrocytic HDAC7 attenuates cognitive impairments in PS19 mice. **A-F** Spatial memory was evaluated using Morris Water Maze (MWM) test at 30 days post-AAV injection. **A** Escape latency to the targeted platform during the training trial. **B** Representative traces of the mice traveled in the in the probe trial after removing the platform. **C** First latency to reach the platform region, **D** exploring time in the targeted zone, (**E**) target crossings and **F** swimming speed in the probe trial. **G** Discrimination index of WT and PS19 mice in the probe trial of Novel Object Recognition (NOR) test. For behavioral tests, *n* = 11 (WT-Vec), 10 (WT-shHD7), 15 (PS19-Vec), 15 (PS19-shHD7) mice per group. **H** Representative images showing neuronal dendrites and spines by Golgi staining in the hippocampus of WT and PS19 mice after AAV injection. Scale bar: 10 μm. **I** Sholl analysis of dendritic intersections. *n* = 28 (WT-Vec), 32 (WT-shHD7), 31 (PS19-Vec), 29 (PS19-shHD7) from 3 mice per group. **J** Quantification of spine numbers, *n* = 33 (WT-Vec), 32 (WT-shHD7), 54 (PS19-Vec), 43 (PS19-shHD7) from 3 mice per group. Statistical significance was determined by two-way ANOVA (A) or one-way ANOVA (C-G and J) or with Tukey’s post hoc analysis. Data are shown as mean ± SEM, *p < 0.05, **p < 0.01, ***p < 0.001, n. s, not significant

Our previous study has revealed that overexpression of HDAC7 in astrocytes induces astrogliosis and neuroinflammation in WT mice [26]. Indeed, we found marked attenuation of astrogliosis and microgliosis in the hippocampus by knockdown of astrocytic HDAC7 in PS19 mice (Supplementary Fig. 2C-F). Given the beneficial effects of astrocytic HDAC7 deficiency on tau pathology, we tested the synaptic and cognitive performance following HDAC7 knockdown. Morris Water Maze (MWM) test showed that HDAC7 knockdown dramatically attenuated spatial learning and memory impairments in PS19 mice (Fig. 3A-E), with the swimming speed unaffected (Fig. 3F). Novel Object Recognition (NOR) test also indicated that HDAC7 knockdown improved the cognition of PS19 mice (Fig. 3G). Analysis of Golgi staining suggested that astrocytic HDAC7 knockdown restored the dendritic complexity and spine numbers of hippocampal neurons in PS19 mice (Fig. 3H-J). Together, these data demonstrated that genetic knockdown of HDAC7 in astrocytes effectively ameliorated synaptic loss and cognitive deficits in PS19 mice.

### Inhibiting HDAC7 by TMP195 alleviates tau pathology and improves cognitive function in PS19 mice

**Fig. 4:**
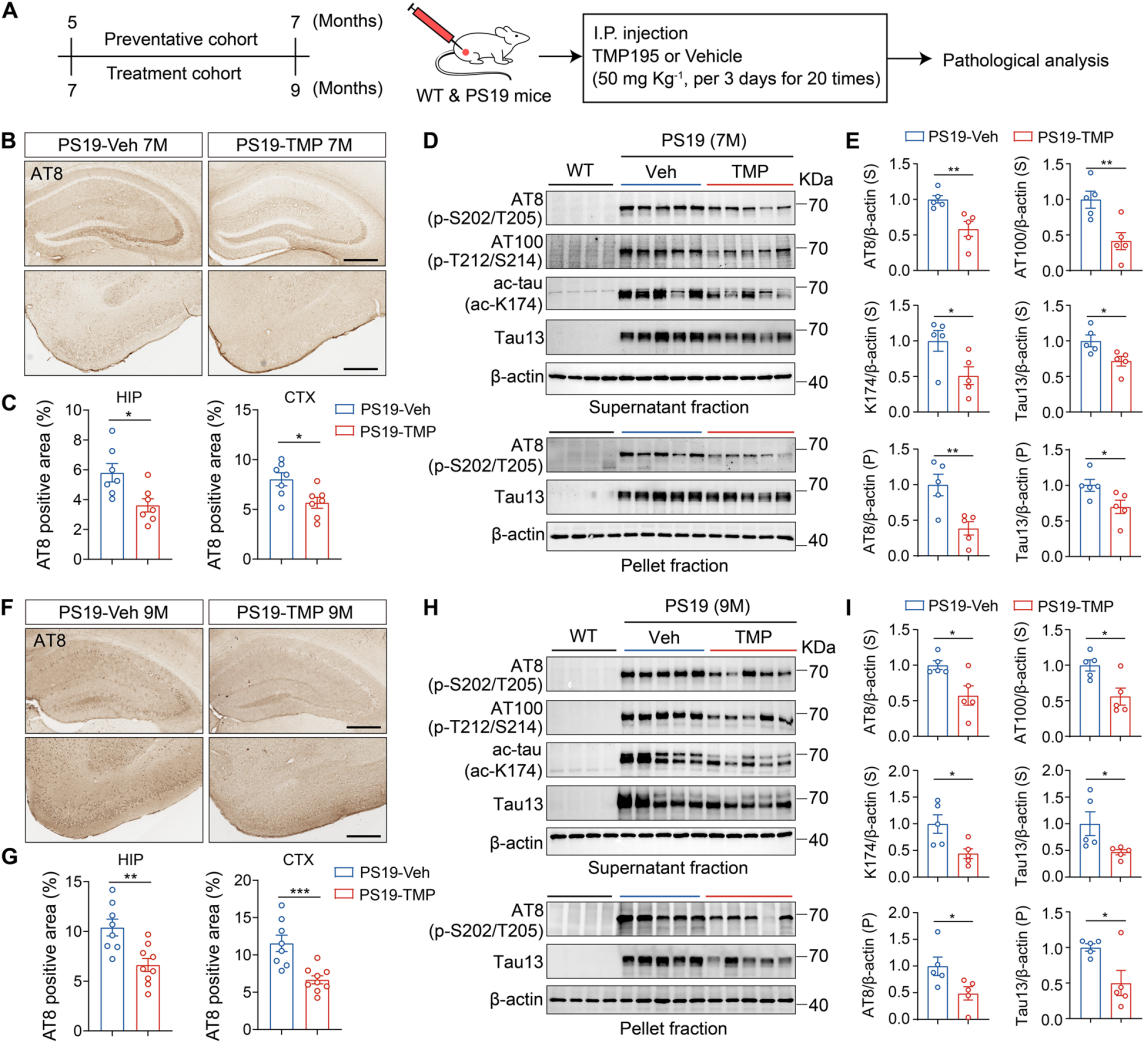
Pharmacological inhibition of HDAC7 diminishes tau pathology in young and aged PS19 mice. **A** Schematic illustration of the in vivo treatment of TMP195 in mice. 5 and 7-month-old mice were grouped into preventative and treatment cohort respectively, and received intraperitoneal injection of TMP195 every 3 days. After 2 months, pathological analysis was performed. **B**, **C** Immunohistochemical staining and quantification of of AT8 in the brain sections of PS19 mice administrated with TMP195 or vehicle in preventative cohort. *n* = 7 mice per group. Scale bar: 200 μm. **D**, **E** Western blotting analysis and quantification of AT8, AT100, PHF13 and tau13 in the supernatant and pellet fraction of hippocampal extracts from WT and PS19 mice in the preventative cohort. *n* = 5 mice per group. **F**, **G** Immunohistochemical analysis of AT8 in the brain sections of PS19 mice in treatment cohort. *n* = 8 (PS19-Veh), 9 (PS19-TMP195) mice. Scale bar: 200 μm. **H**, **I** Western blotting analysis of AT8, AT100, PHF13 and tau13 in the supernatant and pellet fraction of hippocampus from WT and PS19 mice in the treatment cohort. *n* = 5 mice per group. Statistical significance was determined by unpaired Student’s t test. Data are shown as mean ± SEM, *p < 0.05, **p < 0.01, ***p < 0.001

Next, we examined the effects of HDAC7 pharmacological inhibition on tau pathology, synaptic plasticity and cognitive functions. There isn’t known selective inhibitor of HDAC7, but the selective class IIa HDAC inhibitor (TMP195) has been well-characterized to inhibit HDAC7 [26, 30, 31], with reported anti-tumor activities in preclinical studies [32]. Tau pathology in AD progresses gradually and progressively, with neurodegeneration accompanied by astrocytes dysfunction occurring over years. Correspondingly, the elevation of astrocytic HDAC7 becomes evident at 6 months of age and progressing to overt at 9 months of age in PS19 (Fig. 1F, G). Therefore, PS19 and WT mice aged 5 and 7 months were chosen as preventative and treatment cohort respectively, receiving intraperitoneal injection of either TMP195 or a vehicle for two months (50mg kg^−1^, every 3 days) (Fig. 4A). The metabolic curve of intraperitoneally injected TMP195 in the brain was quantified by liquid chromatography-tandem mass spectrometry (LC-MS/MS) (Supplementary Fig. 3A). Of note, hematoxylin-eosin staining in liver, lung, spleen and kidney showed that intraperitoneal injection of TMP195 has little toxicity to peripheral organs (Supplementary Fig. 3B), supporting the safety of TMP195 administration via intraperitoneal injection. In the preventative cohort, immunohistochemical staining with AT8 revealed that TMP195 administration significantly prevented tau accumulation in the brain of PS19 mice (Fig. 4B, C). Tau acetylation regulated by HDACs is known to promote tau accumulation and tau-related neurodegeneration [33]. We observed that the level of acetylated-tau (ac-K174), as well as the levels of AT8, AT100 and tau13, were dramatically reduced in the hippocampus and cortex of PS19 mice after TMP195 administration (Fig. 4D, E and Supplementary Fig. 4A, B). In 9-month-old PS19 mice from the treatment cohort, consistently, a marked reduction of pathological tau levels was detected in the brain of PS19 mice after TMP195 administration, as showed by decreased AT8 staining signals in the brain sections (Fig. 4F, G), and decreased levels of soluble AT8, AT100, ac-K174 and tau13 and insoluble AT8 and tau13 in the extracts of hippocampus and cortex (Fig. 4H, I and Supplementary Fig. 4C, D). Collectively, these results demonstrated that pharmacological inhibition of HDAC7 with TMP195 are efficient to halt tau pathology in both young and aged PS19 mice.

**Fig. 5:**
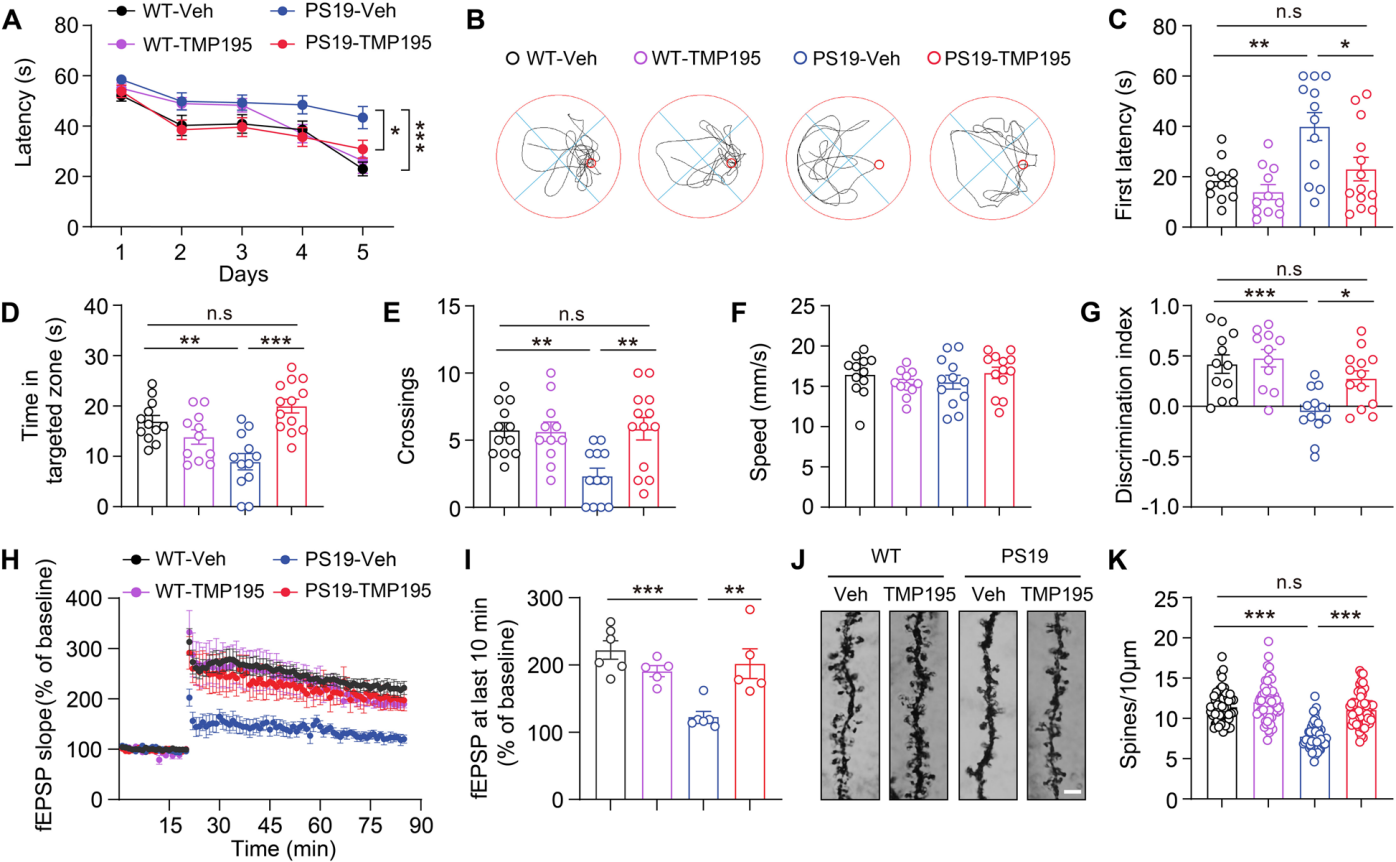
Pharmacological inhibition of HDAC7 ameliorates synaptic and cognitive deficits in PS19 mice. **A-F** Spatial memory was evaluated by MWM test in 7-month-old WT and PS19 mice after TMP195 treatment. **A** Escape latency to the targeted platform during the training trial of MWM. **B** Representative traces of the mice traveled in the in the probe trial after removing the platform. **C** First latency to reach the platform, **D** exploring time in the targeted zone, **E** target crossings and **F** swimming speed in the probe trial. **G** Discrimination index in the probe trial of NOR test of WT and PS19 mice. For behavioral tests, *n* = 12 (WT-Veh), 11 (WT-TMP195), 12 (PS19-Veh), 13 (PS19-TMP195) mice. **H** Slope of field excitatory postsynaptic potential (fEPSP) in response to three trains of high‐frequency stimulation in preventative cohort mice. **I** Quantification of fEPSP slope during the last 10 min recordings in P. *n* = 6 (WT-Veh), 5 (WT-TMP195), 6 (PS19-Veh), 5 (PS19-TMP195) mice. **J**, **K** Golgi staining analysis of dendritic spine numbers in the hippocampus of preventative cohort mice. *n* = 52 (WT-Veh), 55 (WT-TMP195), 51 (PS19-Veh), 61 (PS19-TMP195) from 3 mice per group. Scale bar: 5 μm. Statistical significance was determined by two-way ANOVA (A) or one-way ANOVA (C-I and K) with Tukey’s post hoc analysis. Data are shown as mean ± SEM, *p < 0.05, **p < 0.01, ***p < 0.001, n. s, not significant

In addition, astrogliosis and microgliosis were also attenuated by TMP195 in both preventative and treatment cohort PS19 mice (Supplementary Fig. 5). MWM test showed that TMP195 treatment significantly ameliorated the spatial learning and memory impairments in PS19 mice (Fig. 5A-E), with the swimming speed comparable (Fig. 5F). NOR test also indicated that TMP195 treatment improved memory function in PS19 mice (Fig. 5G). Hippocampal synaptic plasticity, manifested by functional long-term potentiation (LTP) and structural dendritic spines, is the cellular basis of learning and memory [26]. Electrophysiological recordings revealed that the reduced magnitude of hippocampal LTP in PS19 mice was markedly attenuated by TMP195 (Fig. 5H, I). Golgi staining also showed that TMP195 treatment restored the synaptic loss of hippocampal neurons in PS19 mice (Fig. 5J, K).

Taken together, the above results revealed that pharmacological inhibition of HDAC7 with TMP195 can ameliorate synaptic and cognitive impairments and attenuate tau pathology in PS19 mice.

### Inhibition or deletion of HDAC7 enhances tau uptake and degradation in astrocytes

**Fig. 6:**
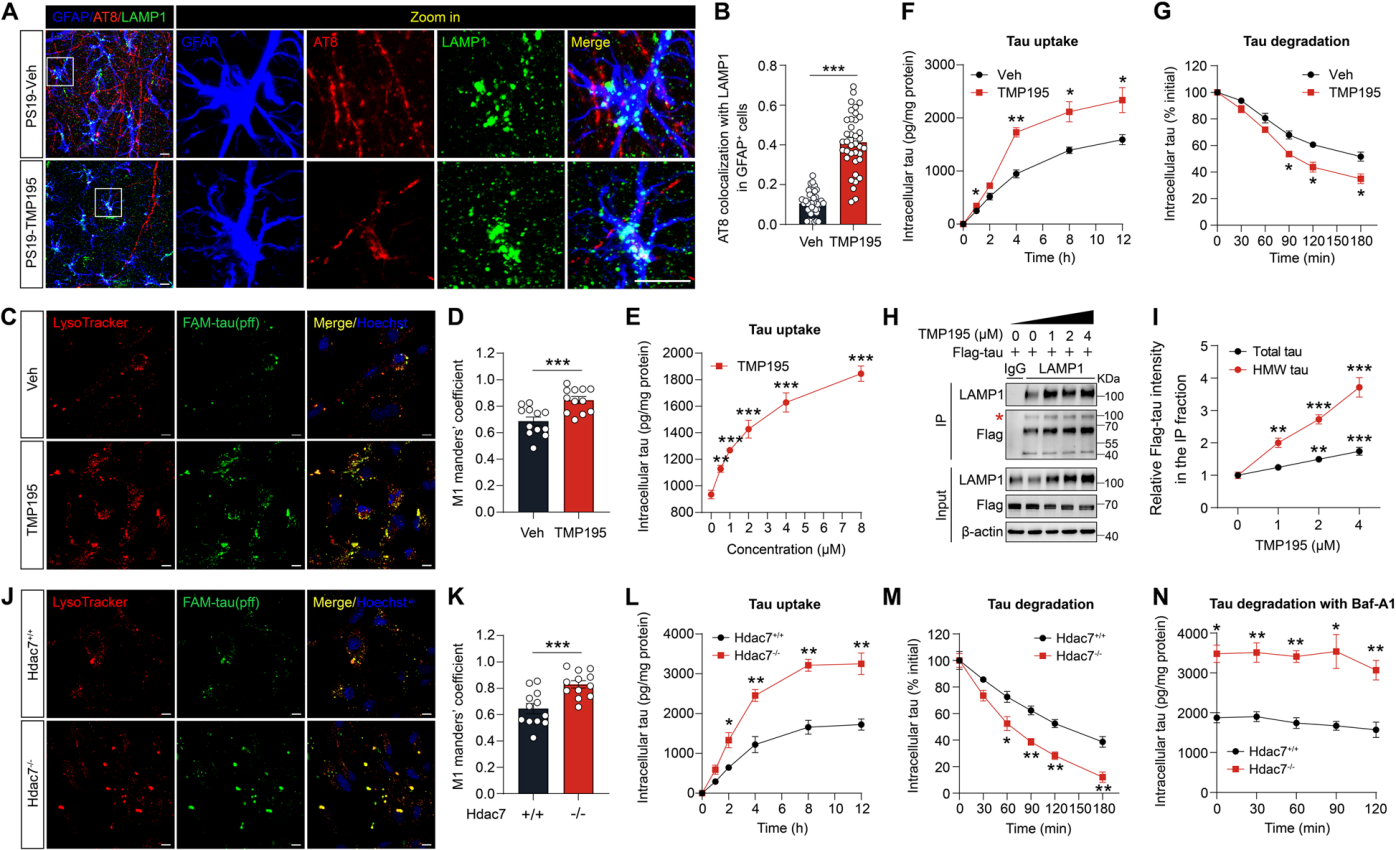
Inhibition or deletion of HDAC7 enhances tau uptake and degradation in astrocytes. **A** Representative immunostaining images of astrocytes (GFAP), phospho-tau (AT8) and lysosomes (LAMP1) in the hippocampus of preventative cohort mice. scale bar: 20 μm. **B** Quantification of engulfed tau in astrocytic lysosomes. *n* = 43 (Veh), 43 (TMP195) cells from 4 mice per group. **C**, **D** Colocalization of tau-pff (FAM-labelled) with Lysotracker Red in primary mouse astrocytes treated with tau-pff (1 μM, this concentration was maintained hereafter) and TMP195 (4 μM) for 4 h. *n* = 12 per group. Scale bar: 20 μm. **E** ELISA analysis of intracellular tau in astrocytes treated with tau-pff and various concentration of TMP195 for 4 h. *n* = 3 per group. **F** ELISA analysis of intracellular tau at various time points in astrocytes treated with tau-pff and TMP195 (4 μM). *n* = 3 per group. **G** Following incubation with tau-pff for 4 h, cells underwent a thorough washing process and were continuously cultured with fresh medium in the presence of TMP195 or vehicle. Intracellular tau was examined at various time points by ELISA. *n* = 3 per group. **H** Co-immunoprecipitation assay showing the interaction of tau and LAMP1 in astrocytes infected with LV-G1-tau(P301S)−3xFlag and then treated with TMP195. **I** Quantification of total and high molecular weight (HMW) tau interacted with LAMP1 in H. *n* = 6 from 3 independent experiments. **J**, **K** Colocalization of tau-pff (FAM-labelled) and Lysotracker Red in control and HDAC7 knockout astrocytes incubated with tau-pff for 4 h. *n* = 12 per group. Scale bar: 20 μm. **L** ELISA analysis of intracellular tau at various time points in control and HDAC7 knockout astrocytes treated with tau-pff. *n* = 3 per group. **M** After incubation with tau-pff for 4 h, cells were thoroughly washed to remove extracellular tau-pff and were continuously cultured with fresh medium. At various time points, intracellular tau levels were quantified by ELISA. *n* = 3 per group. **N** Cells treated as in M with the addition of Bafilomycin A1 (100 nM) for 1 h before washing out tau-pff and cultured in its presence until cells were collected for ELISA analysis. *n* = 3 per group. Statistical significance was determined by unpaired Student’s t test (B, D, F, G and K-N) or one-way ANOVA with Tukey’s post hoc analysis (E and I). Data are shown as mean ± SEM, *p < 0.05, **p < 0.01, ***p < 0.001

Studies of HDAC7 in peripheral immune cells have shown that inhibiting HDAC7 can enhance the phagocytic ability of macrophages, leading to increased anti-tumor activity [30] [32]. Similarly, astrocytes in the brain are rich in genes related to engulfment pathways [34] and are actively involved in the uptake and degradation of tau species released from neurons [7]. Following administration of TMP195, PS19 mice exhibited a significant increase in the colocalization of AT8 positive tau with lysosomes in astrocytes (Fig. 6A, B), indicating improved tau clearance by astrocytes via HDAC7 blockage.

To investigate mechanisms by which HDAC7 inhibition in astrocytes mitigates tau pathology, we examined clearance of tau aggregates in cultured astrocytes. We generated tau-pff using recombinant human tau protein and labelled it with 5-FAM following an established protocol [35]. Primary mouse astrocytes were treated with tau-pff and TMP195, and then co-labelled with Lysotracker Red and Hoechst and imaged using confocal microscopy. Treatment with TMP195 resulted in enhanced tau uptake and lysosomal trafficking in astrocytes (Fig. 6C, D). To confirm the observation, we quantified the level of intracellular tau in cultured astrocytes after treatment of TMP195 with vary concentrations. ELISA analysis of intracellular tau showed that TMP195 increases tau internalization in a concentration dependent manner (Fig. 6E). To test the effects of TMP195 on the kinetics of tau uptake, we incubated astrocytes with tau-pff and TMP195 for increasing periods of time (1, 2, 4, 8 and 12 h) at 37°C. At different time point, ELISA analysis demonstrated that TMP195 treatment significantly increased intracellular tau level compared with controls (Fig. 6F), indicating faster rates of tau internalization by HDAC7 inhibition. Next, we tested the effects of TMP195 on the degradation of internalized tau. Astrocytes were incubated with tau-pff for 4 h and then thoroughly washed. At various time points, intracellular tau level was quantified by ELISA analysis. TMP195 treatment significantly increased the degradation rate of intracellular tau level compared with control cells (Fig. 6G). Furthermore, TMP195 treatment concentration-dependently increased the interaction between tau and lysosomal marker LAMP1 (Fig. 6H, I), as well as the colocalization of tau with LAMP1 (Supplementary Fig. 6A, B). This treatment also reduced tau accumulation in astrocytes overexpressing P301S tau (Supplementary Fig. 6C, D). Collectively, these data demonstrated that HDAC7 inhibition with TMP195 enhances both tau uptake and degradation in cultured astrocytes.

To further confirm that the clearance effects were mediated by HDAC7, we next examined the uptake and degradation of tau in HDAC7 knockout astrocytes. Primary astrocytes from HDAC7^flx/flx^ mice were infected with AAV-GfaABC1D-Cre-NLS to achieve HDAC7 deletion. As expected, deletion of HDAC7 increased colocalization of 5-FAM-labelled tau-pff with Lysotracker Red^+^ signals (Fig. 6J, K). Tau uptake assay revealed that deletion of HDAC7 markedly increased the rate of tau internalization in astrocytes (Fig. 6L). Tau degradation assay revealed that deletion of HDAC7 resulted in the enhanced degradation rate of intracellular tau levels compared with control cells (Fig. 6M). Immunostaining of LAMP1 in tau-overexpressing astrocytes also indicated that HDAC7 deletion increases the colocalization of tau with lysosomes (Supplementary Fig. 6E, F). To ensure whether HDAC7 deletion-mediated clearance of tau proceeded via lysosomal degradation, we blocked lysosomal function in the tau degradation assay using Bafilomycin A1, a H^+^-ATPase inhibitor that impairs lysosomal acidification. Bafilomycin A1 administration almost completely prevented the degradation of internalized tau, in both control and HDAC7 knockout astrocytes (Fig. 6N). Taken together, these data indicated that pharmacological inhibition or genetic deletion of HDAC7 enhances astrocyte-mediated tau clearance, which is dependent on lysosomal function.

### HDAC7 regulates TFEB acetylation and lysosomal biogenesis

**Fig. 7:**
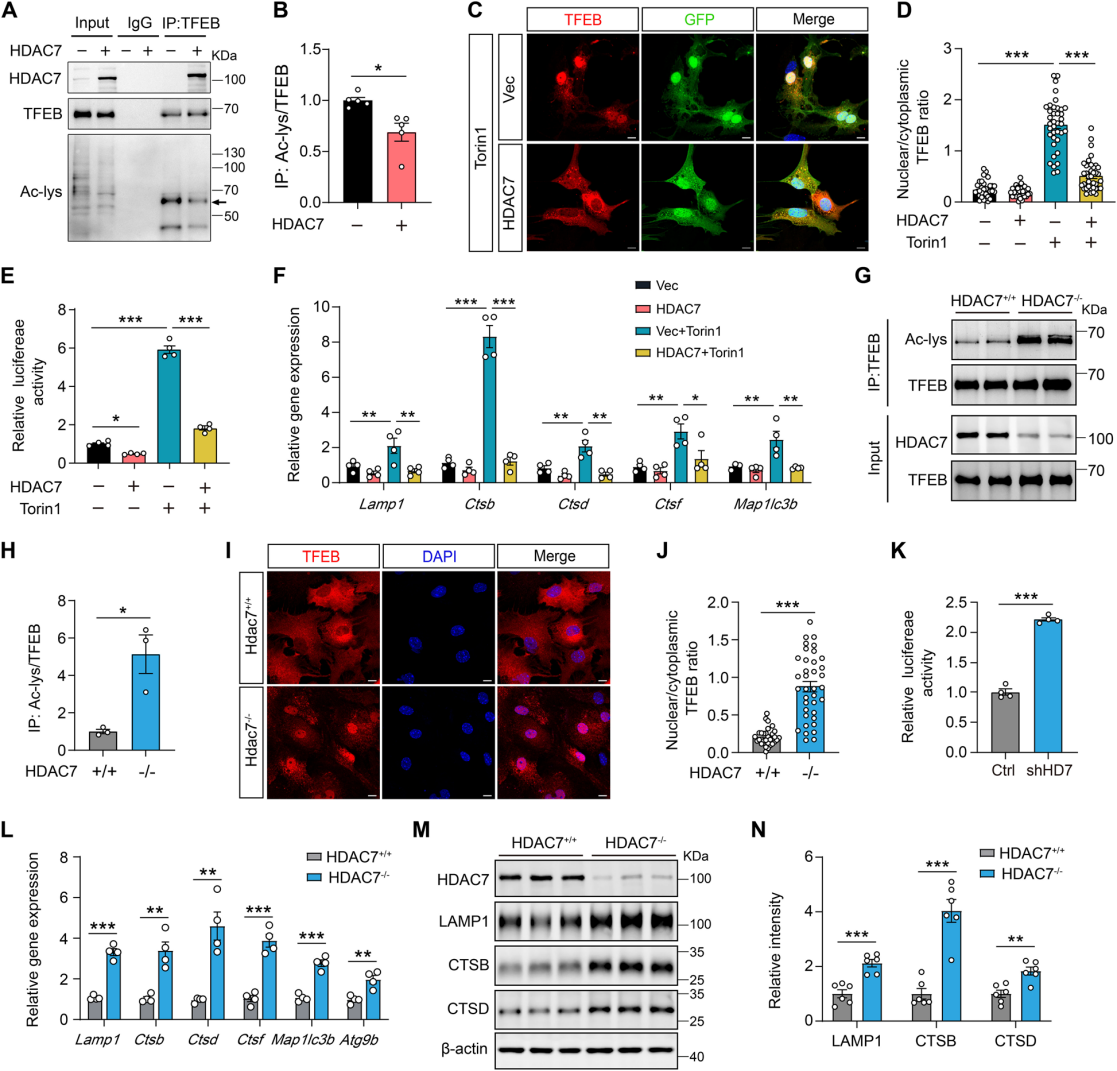
HDAC7 regulates TFEB acetylation and lysosomal biogenesis. **A** Representative blots showing HDAC7-TFEB interaction and TFEB acetylation level by immunoprecipitation conducted in primary astrocytes overexpressing HDAC7 or vector. **B** Quantification of TFEB acetylation in vector and HDAC7-overexpressing astrocytes. *n* = 5 per group. **C** Representative immunostaining images of TFEB in vector and HDAC7-overexpressing astrocytes treated with Torin1 (100 nM) or vehicle for 4 h. Scale bar:10 μm. **D** Quantification of nuclear/cytoplasmic TFEB ratio in C. *n* = 31 (Vec), 32 (HDAC7), 38 (Vec+Torin1), 38 (HDAC7+Torin1) cell per group. **E** Analysis of TFEB transcriptional activity in HEK293T cells transfected with HDAC7 or vector and then treated with Torin1 (100 nM) or vehicle for 4 h. *n* = 4 per group. **F** mRNA levels of Lamp1, Ctsb, Ctsd, Ctsf and Map1lc3b measured by RT-qPCR in cultured astrocytes treated as described in C. *n* = 4 per group. **G**, **H** Immunoprecipitation assay showing the acetylation level of TFEB in WT and HDAC7 knockout astrocytes. *n* = 3 per group. **I** Representative immunostaining images of TFEB in control and HDAC7 knockout astrocytes. Scale bar: 10 μm. **J** Quantification of nuclear/cytoplasmic TFEB ratio in I. *n* = 31 (HDAC7^+/+^), 37 (HDAC7^-/-^). **K** Analysis of TFEB transcriptional activity in HEK293T cells transfected with shRNA targeting HDAC7 (shHD7) or control scramble. *n* = 4 per group. **L** mRNA levels of Lamp1, Ctsb, Ctsd, Ctsf, Map1lc3b and Atg9b quantified by RT-qPCR in control and HDAC7 knockout astrocytes. *n* = 4 per group. **M**, **N** Western blotting analysis and quantification of LAMP1, CTSB and CTSD in control and HDAC7 knockout astrocytes. *n* = 6 per group. Statistical significance was determined by unpaired Student’s t test (B, H, J-L and N) or one-way ANOVA with Tukey’s post hoc analysis (D-F). Data are shown as mean ± SEM, *p < 0.05, **p < 0.01, ***p < 0.001

TFEB plays a critical role in lysosomal biogenesis and functional regulation, and acetylation has been implicated in the regulation of TFEB nuclear translocation and transcriptional activity in the nucleus [28, 36]. Thus, we sought to determine the interaction between HDAC7 and TFEB. Co-immunoprecipitation analysis with TFEB antibody revealed that HDAC7 can directly bind to TFEB and reduce TFEB acetylation level (Fig. 7A and B). It has been observed that TFEB is primarily localized in the cytoplasm of astrocytes under normal culture conditions [7]. To examine whether HDAC7 has an impact on TFEB nuclear translocation, we induced TFEB activation by treating astrocytes with the mammalian target of rapamycin complex 1 (mTORC1) inhibitor Torin1. Interestingly, the nuclear translocation of TFEB in astrocytes induced by Torin1 was almost completely blocked by the overexpression of HDAC7 (Fig. 7C, D). Additionally, overexpression of HDAC7 inhibited the Torin1-induced increase in TFEB luciferase activity (Fig. 7E), and in the transcriptional upregulation of TFEB-targeted lysosomal and autophagic genes (Fig. 7F). Conversely, knockout of HDAC7 resulted in an elevation of TFEB acetylation levels (Fig. 7G, H), leading to the activation of the TFEB-lysosomal pathway, as evidenced by prominent TFEB nuclear translocation (Fig. 7I, J), increased TFEB luciferase activity (Fig. 7K), upregulated mRNA levels of lysosomal and autophagic genes (Fig. 7L), and increased protein levels of lysosomal genes (Fig. 7M, N). Collectively, these findings suggested that HDAC7 plays a critical role in regulating the TFEB-lysosomal pathway involving TFEB acetylation.

Prior studies have demonstrated that TFEB activation is modulated through phosphorylation/dephosphorylation by various upstream kinases, including mTORC1, glycogen synthase kinase-3β (GSK3β), and extracellular signal-regulated kinase (ERK) [14, 37]. Thus, we sought to test the potential involvement of phosphorylation in HDAC7-mediated TFEB signaling. Importantly, both the overexpression and deletion of HDAC7 displayed minimal impacts on the activity of p70 S6 kinase, GSK3β, and ERK1/2 in astrocytes (Supplementary Fig. 7A-D). Furthermore, we constructed Flag-TFEB mutations (S to D) at the S122 and S211 sites to mimic TFEB phosphorylation. Notably, HDAC7 deletion still markedly promoted nuclear translocation of the S122D and S211D mutated TFEB (Supplementary Fig. 7E, F). These findings suggest that HDAC7-mediated acetylation may serve as a novel mechanism for regulating TFEB activity independently of phosphorylation.

### TFEB acetylation at K310 is critical for HDAC7-mediated lysosome biogenesis and tau clearance

**Fig. 8:**
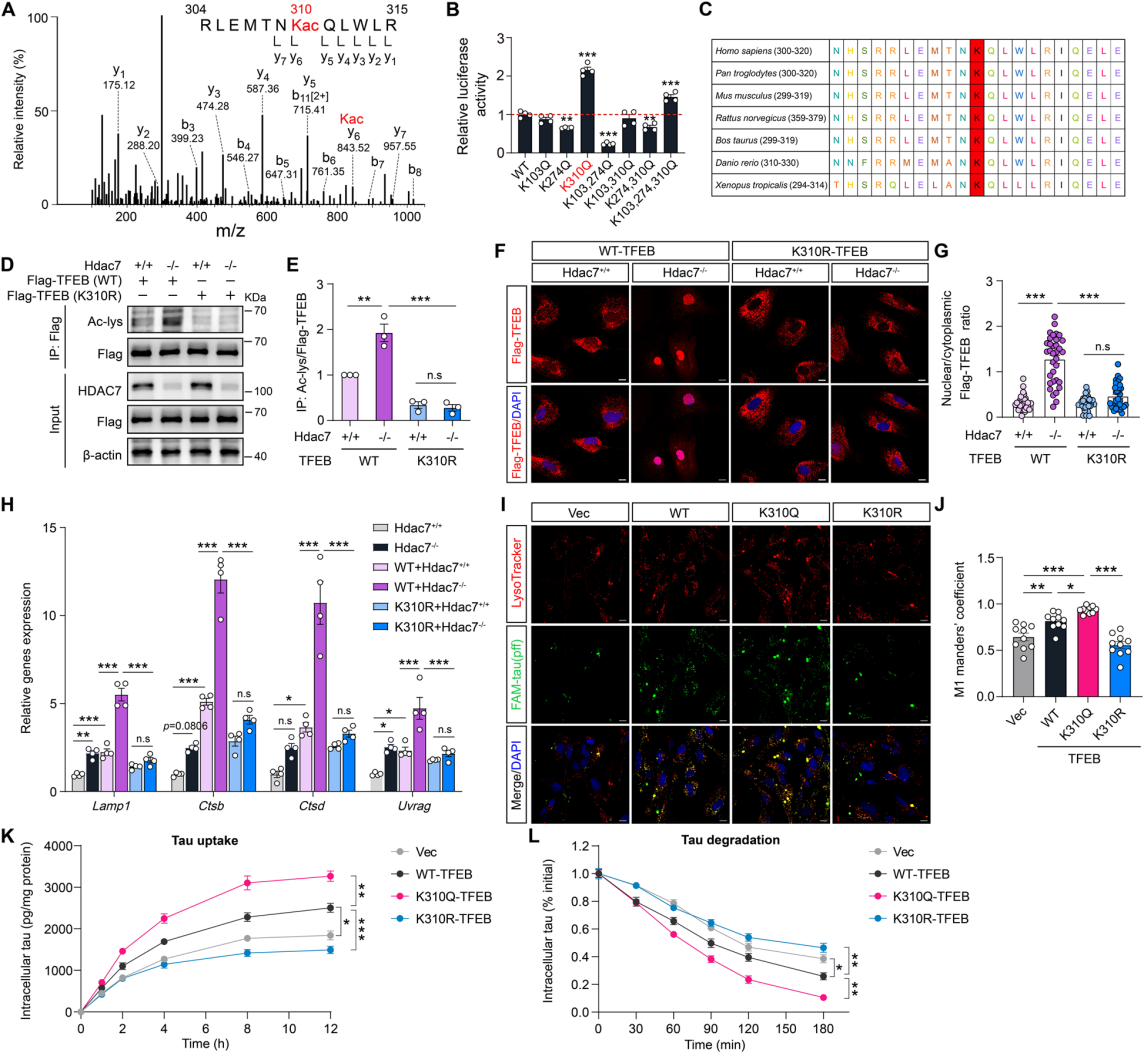
TFEB acetylation at K310 is required for HDAC7 deletion-mediated TFEB nuclear translocation, lysosomal biogenesis and tau clearance in astrocytes. **A** Identification of TFEB acetylation atK310 by mass spectrometry analysis. **B** Quantification of TFEB transcriptional activity in HEK293T cells with different point mutations (K to Q). *n* = 4 per group. **C** Protein sequence alignment of TFEB homologues in different species. The red mark represents the identified K310 residue. **D**,** E** Immunoprecipitation assay showing the acetylation level of exogeneous WT/K310R Flag-TFEB in control and HDAC7 knockout astrocytes. The acetylation level of Flag-TFEB was quantified. *n* = 3 per group. **F** Representative immunostaining images of WT/K310R Flag-TFEB in control and HDAC7 knockout astrocytes. Scale bar: 10 μm. **G** Quantification of Flag-TFEB nuclear/cytoplasmic ratio in F. *n* = 30 (HDAC7^+/+^+WT-TFEB), 34 (HDAC7^-/-^+WT-TFEB), 30 (HDAC7^+/+^+K310R-TFEB), 31 (HDAC7^-/-^+K310R-TFEB). **H** Analysis of Lamp1, Ctsb, Ctsd and Uvrag mRNA levels by qPCR in control and HDAC7 knockout astrocytes treated as indicated. *n* = 4 per group. **I** Representative images showing colocalization of tau-pff and Lysotracker Red in primary astrocytes overexpressed with WT/K310Q/K310R-TFEB or vector. Scale bar = 10 μm. Cells were treated with tau-pff for 4 h and then co-staining with Lysotracker Red. **J** Manders’ coefficient analysis of tau-pff and lysotracker colocalization in I. *n* = 10 per group. **K** Analysis of intracellular tau by ELISA in astrocytes overexpressed with WT/K310Q/K310R-TFEB or vector and then treated with tau-pff for indicated time period. *n* = 3 per group. **L** After incubation with tau-pff for 4 h, tau-pff was removed and cells are continuously cultured for 30–180 min. At various time points, intracellular tau was quantified by ELISA. *n* = 3 per group. Statistical significance was determined by one-way ANOVA with Tukey’s post hoc analysis. Data are shown as mean ± SEM, *p < 0.05, **p < 0.01, ***p < 0.001, n. s, not significant

After determining the direct interaction between HDAC7 and TFEB, we sought to identify the potential TFEB deacetylation site(s) by HDAC7. Flag-TFEB and HDAC7 or control GFP were co-transfected in HEK293T cells and then Flag-TFEB was obtained through immunoprecipitation. After SDS-PAGE, Flag-TFEB was subjected to liquid chromatography and mass spectrometry (LC-MS) analysis. Three acetylation sites on TFEB (K103, K274 and K310) were detected in deacetylated form in HDAC7-overexpressing group (Fig. 8A). Based on the LC-MS results, we generated single (K103Q, K274Q, K310Q), double (K103/274Q, K103/310Q, K274/310Q) and triple (K103/274/310Q) point mutations in TFEB to determine the impact of these acetylation sites on TFEB transcriptional activity. The luciferase activity assay disclosed that the K310Q mutation, but not K103Q or K274Q mutation, resulted in elevated TFEB activity (Fig. 8B), a similar finding that aligns with the HDAC7 knockdown. Sequence alignment of TFEB homologs across a variety of species demonstrated the high degree of conservation of K310 throughout evolution (Fig. 8C). Moreover, K310Q mutation, but not K103Q or K274Q mutation, significantly attenuated HDAC7-induced TFEB deacetylation (Supplementary Fig. 8A, B). These findings provide evidence that HDAC7 might deacetylate TFEB at K310, a conserved acetylation site that has not been identified previously.

To gain a deeper understanding of the role of K310 acetylation in HDAC7-mediated TFEB deacetylation and lysosomal signaling, we conducted an overexpression of WT or K310R mutated Flag-TFEB in HDAC7 knockout astrocytes. The subsequent immunoprecipitation assay, utilizing the Flag antibody, demonstrated that the K310R mutation effectively nullified the increase in TFEB acetylation levels induced by HDAC7 deletion, thereby highlighting the primary role of K310 site in the deacetylation of TFEB prompted by HDAC7 deletion (Fig. 8D, E). Immunostaining analysis using the Flag antibody demonstrated that the K310R mutation obstructed the HDAC7 deletion-induced nuclear translocation of TFEB (Fig. 8F, G). Moreover, mRNA analysis indicated that K310R mutation attenuated the upregulation of TFEB target genes following HDAC7 deletion observed in the WT-TFEB group (Fig. 8H). Interestingly, K310R mutation disrupted Torin1-induced TFEB nuclear translocation, suggesting that acetylation at K310 represents an essential event for TFEB activation independently of its dephosphorylation (Supplementary Fig. 8C, D). Further, the influence of the K310Q mutation on TFEB activity was further investigated in astrocytes. The K310Q mutation has been observed to restore the acetylation level of TFEB (Supplementary Fig. 8E, F), facilitate TFEB nuclear translocation (Supplementary Fig. 8G, H), and reestablish the expression of TFEB target genes (Supplementary Fig. 8I) in HDAC7-overexpressing cells. Taken together, these findings provide persuasive evidence that the acetylation of TFEB at K310 plays a crucial role in the modulation of the TFEB-lysosomal pathway mediated by HDAC7.

Next, we investigated the influence of TFEB K310 acetylation on the elimination of extracellular tau aggregates. Primary astrocytes were overexpressed with WT-TFEB, K310Q-TFEB, K310R-TFEB or a control vector, followed by the treatment with tau-pff. Fluorescence staining with Lysotracker Red unveiled that WT-TFEB facilitated the colocalization of tau-pff with lysosomes in comparison to the control vector. The K310Q mutation amplified this effect, while the K310R mutation hindered it (Fig. 8I, J). In the tau uptake and degradation assay, analysis of intracellular tau indicated that overexpression of WT-TFEB resulted in a slight augmentation of tau uptake and degradation (Fig. 8K, L). These effects were significantly amplified by the overexpression of the K310Q mutated TFEB, but were inhibited by the overexpression of the K310R mutated TFEB (Fig. 8K, L). These findings imply that the acetylation of TFEB at K310 is both necessary and sufficient for facilitating the clearance of tau by astrocytes.

### HDAC7 inhibition restores TFEB K310 acetylation in PS19 mice

**Fig. 9:**
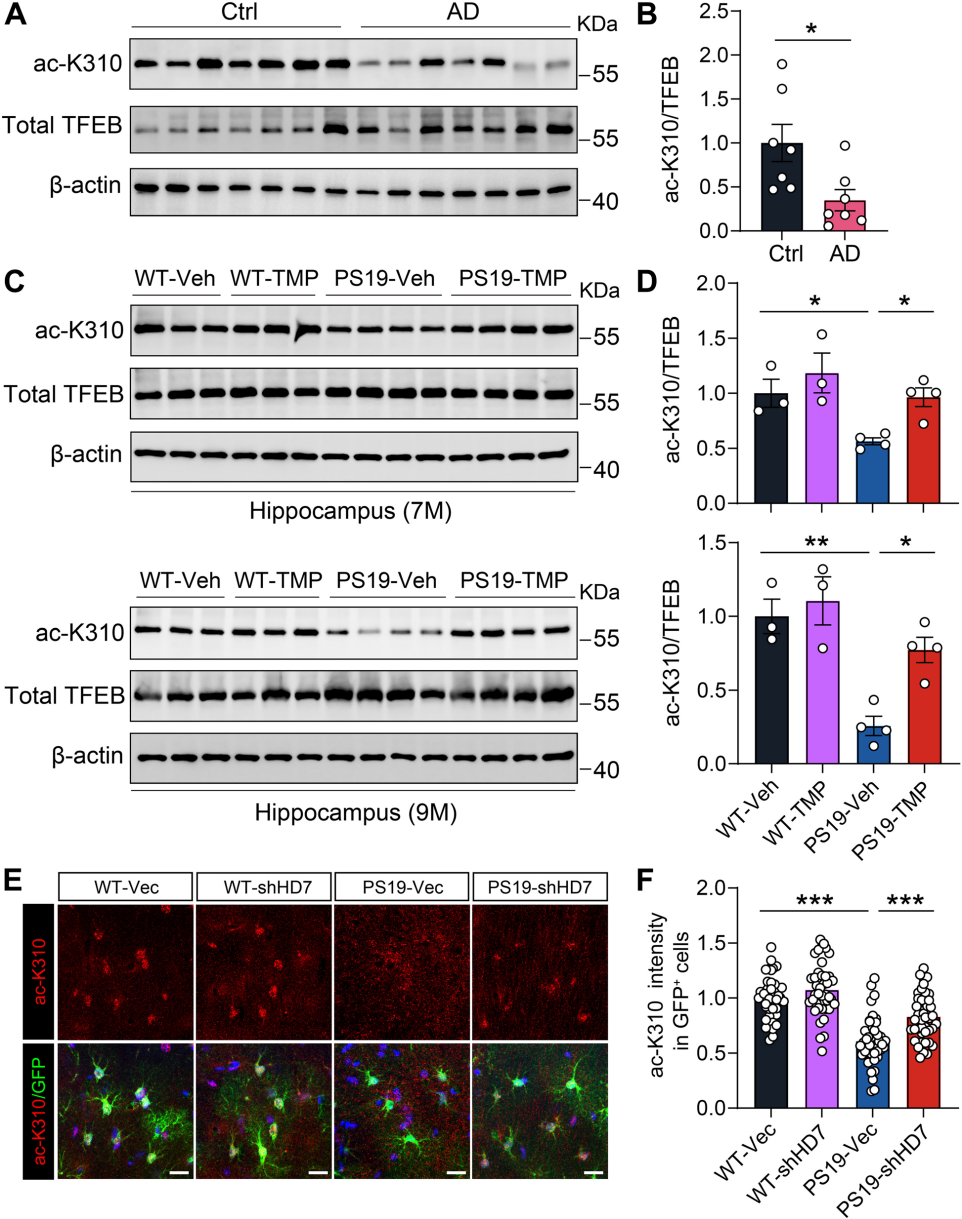
TFEB acetylation at K310 is reduced in AD patients and PS19 mice and inhibiting HDAC7 can restore TFEB acetylation in PS19 mice. **A**, **B** Western blotting analysis and quantification of ac-K310 and total TFEB in the brain lysates of AD patients and control individuals. *n* = 7 per group. **C**, **D** Western blotting analysis and quantification of ac-K310 and total TFEB in the hippocampus of 7- and 9-month-old WT and PS19 mice treated with TMP195 or vehicle. *n* = 3 (WT-Veh), 3 (WT-TMP195), 4 (PS19-Veh), 4 (PS19-TMP195). **E** Representative immunostaining images of ac-K310 in the hippocampus of WT and PS19 mice injected with AAV-G1-shHDAC7-GFP (shHD7) or AAV-G1-GFP. Scale bar = 20 μm. **F** Quantification of ac-K310 intensity in GFP^+^ cells. *n* = 39 (WT-Vec), 42 (WT-shHD7), 42 (PS19-Vec), 43 (PS19-shHD7). Statistical significance was determined by unpaired Student’s t test (B) or one-way ANOVA with Tukey’s post hoc analysis (D and F). Data are shown as mean ± SEM, *p < 0.05, **p < 0.01, ***p < 0.001, n. s, not significant

To characterize the involvement of TFEB K310 acetylation in AD and tauopathies, a rabbit polyclonal antibody, ac-K310, was generated using an antigen containing acetyl-K310. Overexpression of HDAC7 led to a significant decrease in ac-K310 signals in primary astrocytes, demonstrating the specificity of the antibody (Supplementary Fig. 9). Subsequently, the levels of ac-K310 and total TFEB were measured in AD patients and PS19 mice. Importantly, western blot analysis revealed a marked decrease in ac-K310 levels in postmortem brain tissues of AD patients compared to controls, while total TFEB levels remained unchanged (Fig. 9A, B). Consistent with the findings in AD patients, a reduction in the level of ac-K310 was observed in the hippocampus of both 7- and 9-month-old PS19 mice compared to age-matched WT mice, and treatment with TMP195 restored ac-K310 levels in PS19 mice (Fig. 9C, D). Furthermore, immunostaining of ac-K310 showed that the decreased ac-K310 levels in astrocytes of PS19 mice was markedly improved by HDAC7 knockdown (Fig. 9E, F). These findings highlight the importance of TFEB K310 acetylation in the beneficial outcomes associated with HDAC7 inhibition in PS19 mice.

## Discussion

In the present study, we report that HDAC7 is a key regulator of lysosomal clearance in astrocyte-mediated tau clearance. Pharmacological inhibition or knockdown of HDAC7 enhances tau uptake and lysosomal degradation by astrocytes, leading to reduced tau pathology and improved synaptic and cognitive functions in PS19 mice. In cultured astrocytes, HDAC7 controls TFEB-dependent lysosomal biogenesis and tau clearance through directly deacetylating TFEB at a newly identified site, K310. Ac-K310 is decreased in astrocytes of PS19 mice and AD patients, and can be restored by HDAC7 inhibition or knockdown. Our findings highlight the significance of astrocytes in the removal of tau protein via the HDAC7-TFEB lysosomal pathway, indicating its potential as a therapeutic target for the treatment of AD and other tauopathies.

HDACs are potential therapeutic targets in neurodegenerative diseases including AD [38], but few references report the linkage between IIa HDACs and tauopathies. In the current study, we focused on class IIa HDACs and observed that HDAC7 was age-dependently elevated in activated astrocytes in PS19 mice and AD patients, with other three class IIa HDACs unchanged. Class IIa HDACs undergo nucleocytoplasmic shuttling to exert regulatory effects on neuronal function and neurodegeneration. HDAC4 and HDAC5 are mislocalized to the nucleus in neurons from Parkinson’s disease (PD) models, and their pharmacological inhibition show protection on PD-related neurodegenerative features [23, 39]. In AD, HDAC4 is upregulated in the nucleus of ApoE4 astrocytes and its inhibition rescues Aβ clearance deficits by astrocytes [24]. But the in vivo effects haven’t been investigated. Differently, we found elevated HDAC7 mainly in the cytoplasm but not nucleus of astrocytes in PS19 mice and AD patients (as seen by its colocalization with cytoskeleton protein GFAP). Our result was also in accordance with previous study which revealed that increased HDAC7 mainly locates in the cytoplasm of astrocytes upon LPS stimulation [25], suggesting a cytoplasmic specific function of HDAC7 in pathological processes. Our findings demonstrate that HDAC7 inhibition results in increased presence of AT8-positive tau in astrocytic lysosomes in vivo and HDAC7-deficient astrocytes exert increased uptake of tau-pff in vitro, suggesting that HDAC7 deficiency may enhance astrocyte phagocytosis. Indeed, studies in the peripheral immune cells have revealed that HDAC7 inhibition or knockdown promotes macrophage phagocytosis under pathological and physiological conditions. HDAC7 is highly expressed in pre-B cells and down-regulated during their conversion to macrophages, while re-expression of HDAC7 in macrophages suppresses the phagocytic function through transcriptional silencing phagocytosis-related genes [40]. In macrophages from chronic lymphocytic leukemia patients, HDAC7 knockdown or inhibition enhances macrophages phagocytic responses by activating Bruton’s tyrosine kinase [30]. In breast cancer model, HDAC7 inhibition with TMP195 induces recruitment and differentiation of phagocytic and stimulatory macrophages, resulting in enhanced antitumor efficiency [32]. In the brain, although microglia are the professional phagocytes, our observations in PS19 mice reveal that HDAC7 rarely expresses in microglia, consistent with its expression pattern reported by single nuclear RNA-seq in AD patients [41]. Thus, HDAC7 is an astrocyte lineage specific regulator of phagocytic function in the brain. However, the molecular mechanisms underlying the enhanced phagocytosis in HDAC7 deficient astrocytes remain unknown, maybe involving heparan sulfate proteoglycans-mediated macropinocytosis [7] or potential tau receptors such as integrin αV/β1 receptor [35]. Further investigation is needed to address this issue.

Astrocytes are enriched of phagocytic genes and possess the capability to act as phagocytes in response to brain injury or neurodegenerative conditions [34, 42]. In the context of tauopathy, while tau primarily forms intraneuronal aggregates, accumulating evidence from human studies and disease models suggests that pathological tau propagation occurs via cell-to-cell transmission [43–46]. In vitro study demonstrates that astrocytes can readily internalize and degrade extracellular tau [7]. It is important to note that astrocytes may exhibit lower efficiency in degrading protein aggregates compared to microglia, potentially leading to the resecretion and propagation of pathologies. Our findings demonstrate that HDAC7 deficiency improves tau elimination efficiency in cultured astrocytes and reduces tau accumulation in mice following HDAC7 knockdown or inhibition, indicating that HDAC7 deficiency-induced astrocyte clearance may effectively mitigate tau pathology. It has been shown that an excess of tau in astrocytes can lead to the activation of NF-κB and the transformation of neurotoxic reactivity, exacerbating neuronal dysfunction [35, 47]. Studies in inflammatory astrocytes and macrophages have revealed that downregulation or inhibition of HDAC7 can prevent NF-κB activation and inflammatory responses induced by LPS [26, 48], suggesting that suppressing HDAC7 is an anti-inflammatory strategy for tau clearance. Our study also found that reducing or inhibiting HDAC7 can alleviate synaptic impairments and cognitive deficits in PS19 mice, providing further support for the therapeutic potential of HDAC7 suppression in promoting astrocyte-mediated clearance of tau pathology.

Previous studies have established that the activation of TFEB-mediated lysosomal biogenesis enhances astrocyte-mediated clearance, thus attenuating neurodegeneration. Exogenous overexpression of TFEB has been shown to enhance the uptake and lysosomal degradation of tau-pff and Aβ oligomers in astrocytes, leading to reduced tau pathology and spreading in P301S tau mice [7], as well as decreased amyloid plaque formation in APP/PS1 mice [16]. Conversely, the deletion of TFEB has been found to impede the uptake of tau by astrocytes [7]. However, the specific regulatory mechanisms necessary to enhance astroglial TFEB activity in AD remain unclear. A large body of literature has been dedicated to the study of dephosphorylation-mediated TFEB activation. In this study, we demonstrate that TFEB acetylation, induced by HDAC7 deletion, facilitates the nuclear translocation of both WT and phospho-mimic mutated TFEB, and lysosomal biogenesis without affecting its upstream phosphokinase activity. Conversely, TFEB deacetylation by HDAC7 leads to cytoplasmic retention, which is resistant to mTORC1 inhibition. Moreover, the deacetylated TFEB failed to move into the nucleus in response to mTORC1 inhibition. These suggest that the acetylation-dependent regulation of TFEB activity by HDAC7 may represent a novel mechanism independent of TFEB dephosphorylation. Our results are in accordance with previous findings in other cell systems, indicating that increased TFEB acetylation by inhibiting HDAC with pan HDAC inhibitors SAHA or TSA, or silencing class IIa HDAC5 and HDAC9, leads to TFEB activation and enhanced lysosomal biogenesis independent of TFEB dephosphorylation [28, 36]. Despite we have established the direct interaction between TFEB and HDAC7, the potential involvement of other molecules in this regulatory mechanism cannot be ruled out. For example, 14-3-3 proteins are known to interact with TFEB, sequestering it in the cytoplasm by recognizing Ser211-phosphorylated TFEB [49]. HDAC7 has been shown to interact with multiple 14-3-3 proteins [50] and modulate their interactions with binding partners [51]. Consequently, it is possible that 14-3-3 proteins may similarly recognize the K310 deacetylated TFEB, thereby contributing to the HDAC7-mediated cytoplasmic retention of TFEB. This hypothesis warrants further investigation.

Previous studies have identified multiple acetylation sites on TFEB that play a role in regulating its activity, including K91, K103, K116, K236, K237, K430 and K431 [28, 36]. However, following overexpression of HDAC7, only K103 was detected in a deacetylated form among these sites. The K103 had no apparent effect on TFEB activity, a finding undocumented previously. Instead, we identified that acetylation of TFEB at a novel K310 site appears to be critical for promoting TFEB nuclear translocation and transcriptional activity. Remarkably, a K310 acetylation mimic mutant (K310Q) of TFEB showed enhanced nuclear translocation and was resistant to HDAC7 overexpression, while the opposite K310 acetyl-dead mutation (K310R) retained TFEB in cytoplasm even after HDAC7 deletion, thus demonstrating a key requirement for K310 acetylation in driving TFEB activation and lysosomal biogenesis. Moreover, K310Q mutated TFEB was more efficient in driving tau uptake and lysosomal degradation for astrocytes, while K310R mutation almost completely blocked it, suggesting that K310 acetylation is a functional determination of TFEB-mediated tau clearance. Different from our findings on K310, Bao et al have demonstrated that deacetylation of TFEB at K116 by SIRT1 facilitates fibrillar Aβ degradation by upregulating lysosomal biogenesis in microglia [52]. However, we showed here that HDAC7 has few effects on K116 acetylation by LC-MS analysis, suggesting that different HDACs may act on distinct TFEB acetylation sites and result in diverse effects on AD pathology. The specific acetyltransferase(s) responsible for promoting TFEB K310 acetylation remain unidentified, and the potential involvement of other deacetylase modifiers and proteins at this site is still unknown. Further research in these areas will enhance our comprehension of the role of this acetylation site in regulating TFEB activity.

Our findings also reveal the importance of TFEB K310 acetylation in tau pathology in vivo. Reduced levels of ac-K310 were observed in AD patients and 7-mon-old PS19 mice, and even worse in 9-mon-old PS19 mice, consistent with the alternations of its upstream deacetylase HDAC7. HDAC7 inhibition regained ac-K310 levels in both preventative and treatment cohort PS19 mice, combined with the attenuated tau pathology by HDAC7 intervention, emphasizing the functional importance of ac-K310 in the protection of tauopathy. Moreover, the restored ac-K310 levels in astrocytes by HDAC7 knockdown in PS19 mice provide further evidence supporting the requirement of ac-K310 in boosting astrocytic clearance function. Thus, therapies designed to induce astrocyte lysosomal clearance in disease pathogenesis via manipulation of HDAC7 or its downstream target ac-K310 on TFEB may be advantageous for preventing AD and other neurodegenerative diseases.

## Conclusions

In summary, HDAC7 is increased in the astrocytes of AD patients and PS19 mice, and it could induce AD-like tau pathologies via deacetylating TFEB and inhibiting lysosomal biogenesis in astrocytes. Downregulating astrocytic HDAC7-TFEB lysosomal signaling yields promising results in boosting tau clearance, diminishing tau pathology, and ameliorating synaptic plasticity and cognitive function. Our study not only reveals a novel mechanism underlying tau accumulation, but also provides promising therapeutic targets for AD and other tauopathies.

## Supplementary Information

Additional file 1.

## Data Availability

All data and resources that support the findings of this study are available from the corresponding author upon reasonable request.

## Abbreviations

NFTs: Neurofibrillary tangles
AD: Alzheimer’s disease
Aβ: Amyloid-β
HDAC: Histone deacetylase
TFEB: Transcriptional factor EB
PSP: Progressive supranuclear palsy
Tau-pff: Tau preformed fibrils
AAV: Adeno-associated virus
LV: Lentivirus
Cre: Cre-recombinase
NLS: Nuclear localization signal
ShHD7: Small hairpin RNA targeting HDAC7
ELISA: Enzyme-linked immunosorbent assay
RT-qPCR: Real time quantitative PCR
ApoE4: Apolipoprotein E4
LC-MS: Liquid chromatography-tandem mass spectrometry
NOR: Novel object recognition
MWM: Morris Water Maze
PD: Parkinson’s disease
LPS: Lipopolysaccharide
SIRT1: Sirtuin 1
NF-κB: nuclear factor-κB
CTSB: Cathepsin B
CTSD: Cathepsin D
LAMP1: Lysosome-associated membrane protein 1
mTORC1: Mammalian target of rapamycin complex 1
GSK3β: Glycogen synthase kinase-3β
ERK1/2: Extracellular regulated kinase 1/2
GFAP: Glial fibrillary acidic protein
Iba1: Ionized calcium binding adapter molecule 1
NeuN: Neuronal nuclei
TSA: Trichostatin A
SAHA: Suberoylanilide hydroxamic acid
