# Supplementary material for: Upregulated astrocyte HDAC7 induces Alzheimer-like tau pathologies via deacetylating transcription factor-EB and inhibiting lysosome biogenesis
Source: Mol Neurodegener. 2025 Jan 13;20:5. doi: 10.1186/s13024-025-00796-2 (PMC11727263; doi:10.1186/s13024-025-00796-2)
Supplement: Supplementary file 1 — Additional file 1. [file 13024_2025_796_MOESM1_ESM.docx]

**Supplementary information**

**Supplementary Figure 1**


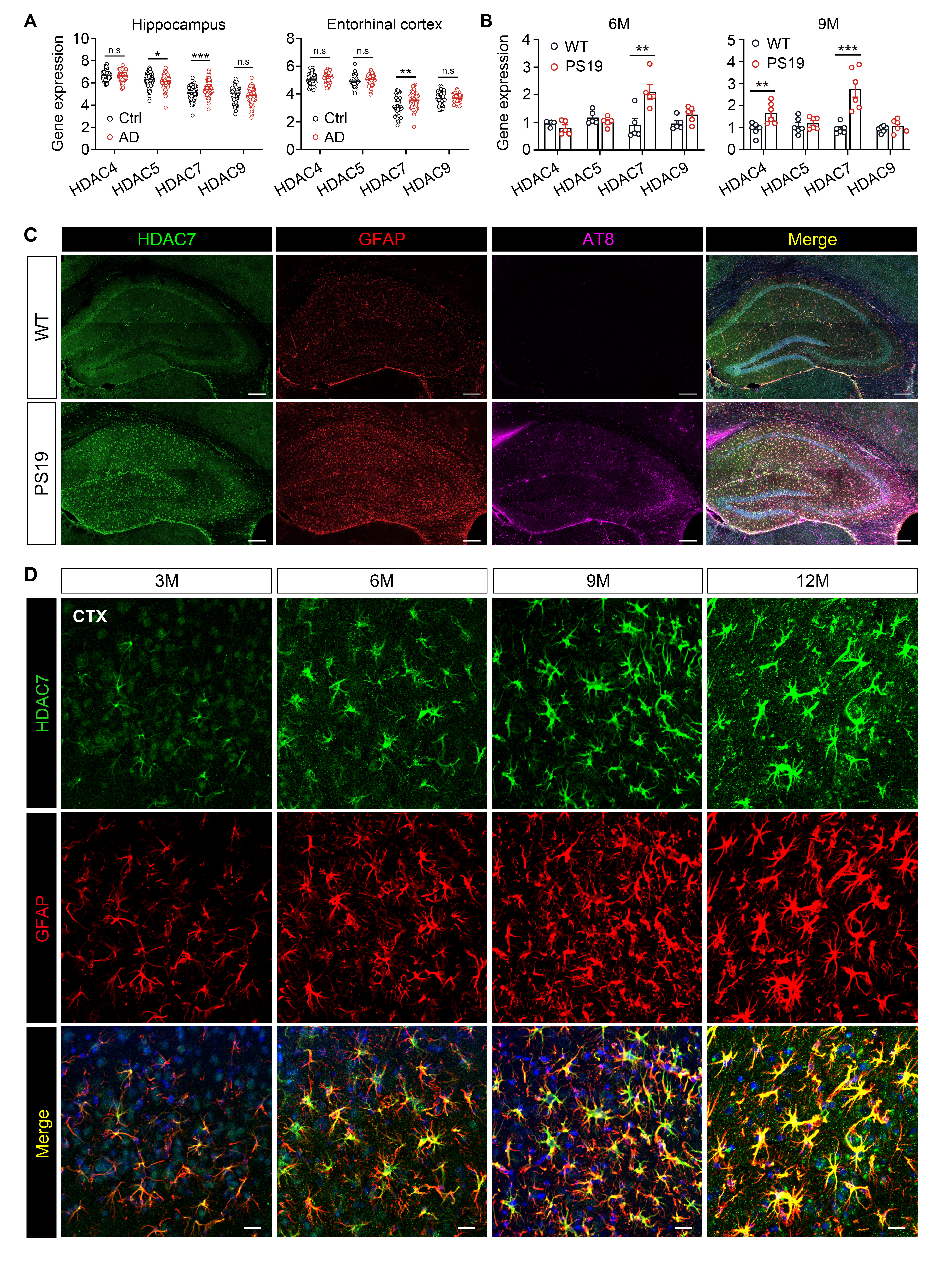


**Supplementary Figure 1 The mRNA expression of class IIa HDACs (HDAC4, 5, 7 and 9) and protein expression of HDAC7 in PS19 mice.**

(**A**) Gene levels of Class IIa HDACs (HDAC4, 5, 7 and 9) in the hippocampus and entorhinal cortex of AD patients and control individuals. All data are obtained from Alzdata database (http://www.alzdata.org). hippocampus: n = 66 (Ctrl), 74 (AD), entorhinal cortex: n = 39 (Ctrl), 39 (AD). (**B**) mRNA levels of HDAC4, 5, 7 and 9 measured by RT-qPCR in the hippocampus of 6- and 9-month-old WT and PS19 mice n = 5 (6M group), 6 (9M group). (**C**) Co-immunostaining of HDAC7, GFAP and AT8 in the hippocampus of 9-month-old WT and PS19 mice. Scale bar: 200 μm. (**D**) Representative immunostaining images of HDAC7 and GFAP in the cortex of 3, 6, 9 and 12-month-old PS19 mice. Scale bar: 20 μm. Statistical significance was determined by unpaired Student’s t test. Data are shown as mean ± SEM, **p* < 0.05, ***p* < 0.01, ****p* < 0.001, n. s, not significant.

**Supplementary Figure 2**


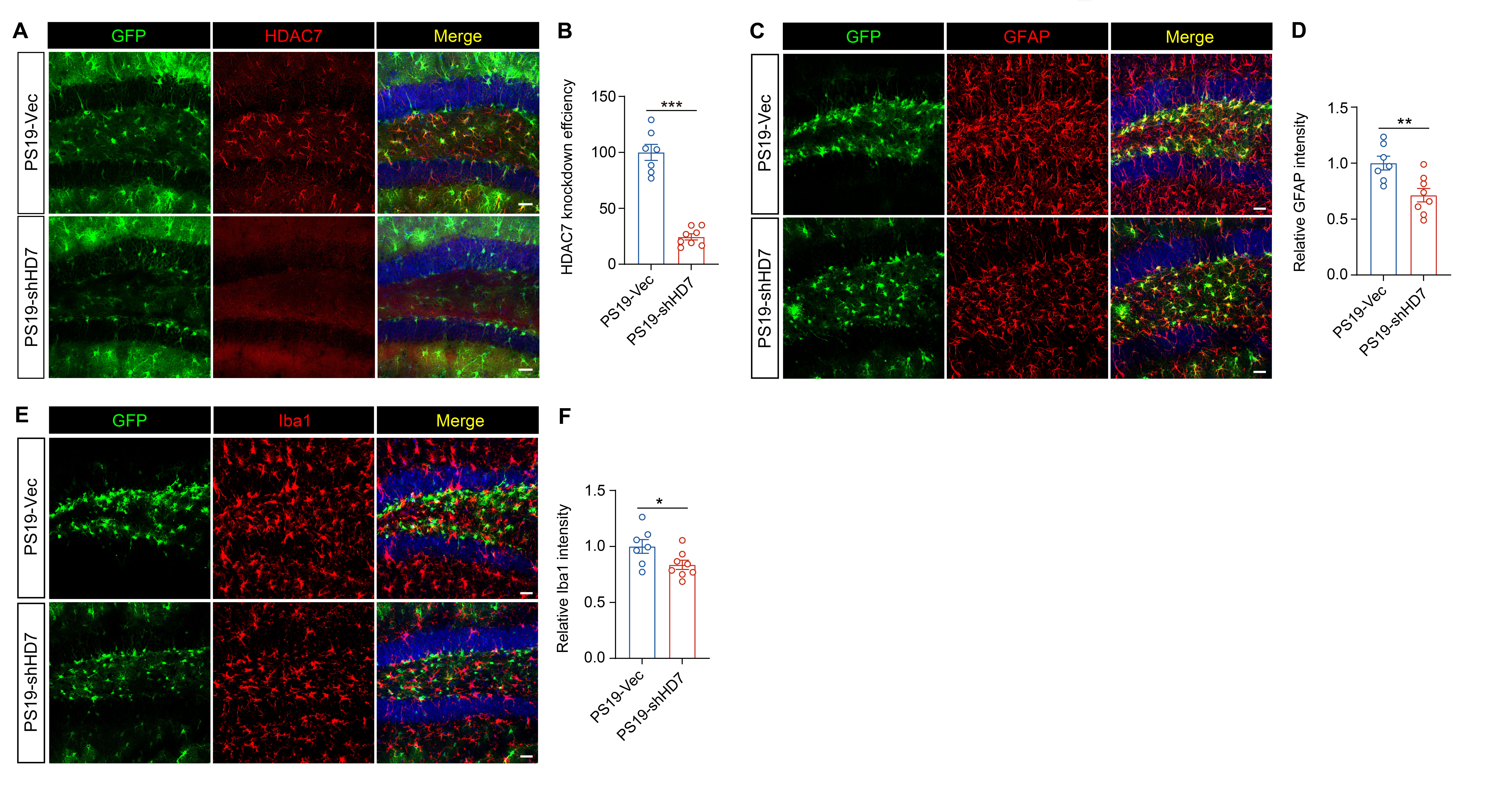


**Supplementary Figure 2 Immunostaining of NeuN and HDAC7 in PS19 mice injected with AAVs.**

(**A, B**) Representative immunostaining images and quantification of HDAC7 knockdown efficiency in the hippocampus of PS19 mice after AAV injection. n = 7 (PS19-Vec), 8 (PS19-shHD7). Scale bar: 40 μm. (**C, D**) Representative immunostaining images and quantification of GFAP in the hippocampus of PS19 mice after AAV injection. n = 7 (PS19-Vec), 8 (PS19-shHD7). Scale bar: 40 μm. (**E, F**) Representative immunostaining images and quantification of Iba1 in the hippocampus of PS19 mice after AAV injection. n = 7 (PS19-Vec), 8 (PS19-shHD7). Scale bar: 40 μm. Statistical significance was determined by unpaired Student’s t test. Data are shown as mean ± SEM, **p* < 0.05, ***p* < 0.01, ****p* < 0.001.

**Supplementary Figure 3**


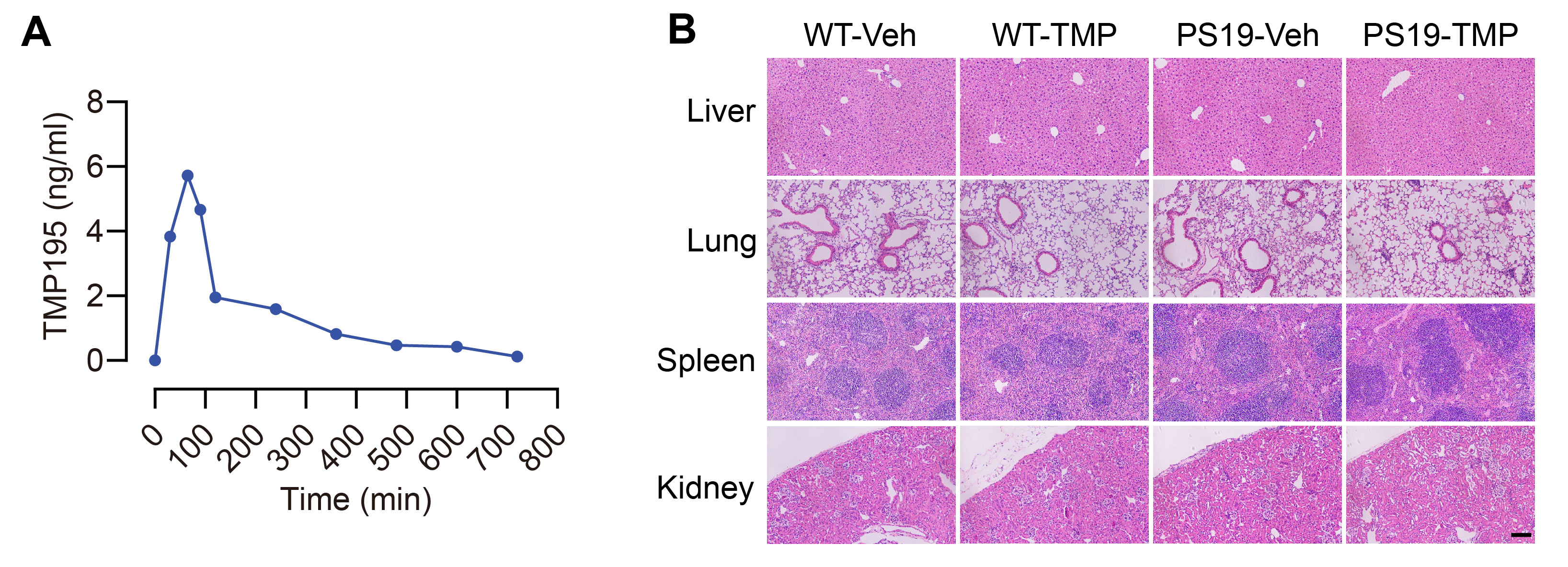


**Supplementary Figure 3 TMP195 enters the brain and shows little toxicity to** **peripheral organs.**

(**A**) Metabolic time curve of TMP195 in the brain of C57/BL6 mice using mass spectrometry analysis. 5-month-old C57BL/6 mice were intraperitoneally injected with TMP195 (50 mg/kg) and sacrificed at 2 h, 4 h, 6 h, 8 h, 10 h or 12 h post injection. The concentration of TMP195 in mice brains were extracted by methanol and quantified using mass spectrometry analysis. n = 1 mouse per time point. (**B**) hematoxylin-eosin staining analysis in the liver, lung, spleen and kidney tissues of WT and PS19 mice treated with TMP195 or vehicle. Scale bar: 200 μm.

**Supplementary Figure 4**


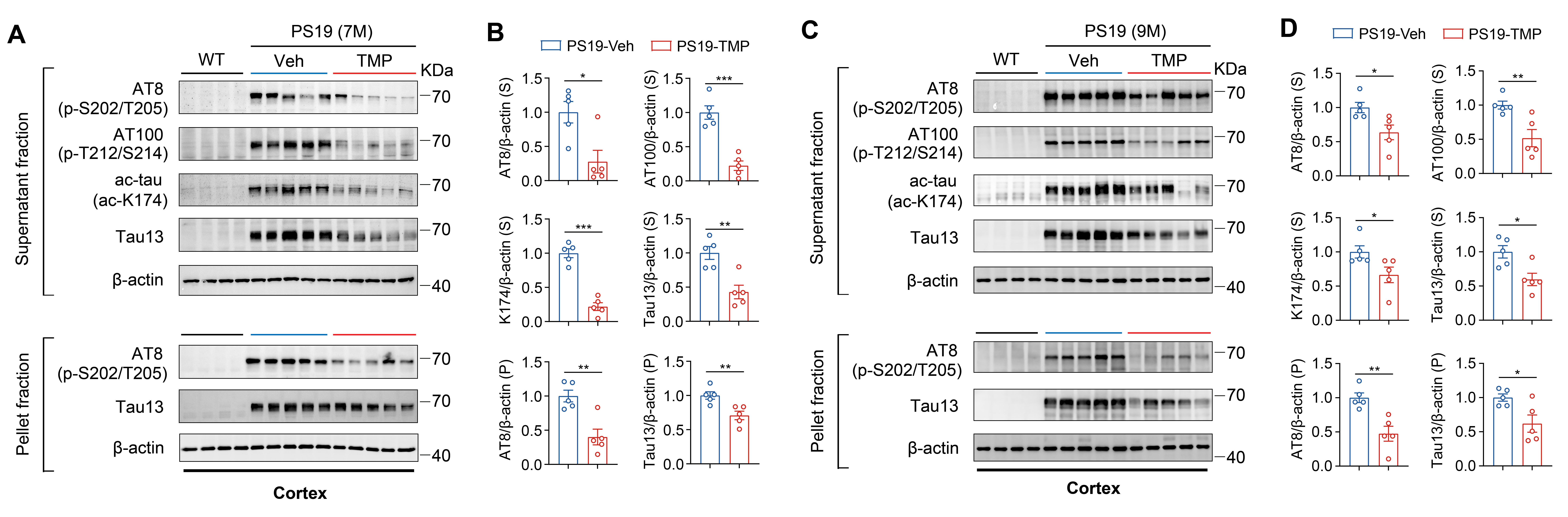


**Supplementary Figure 4 TMP195 treatment reduces tau accumulation in the cerebral cortex of both young and aged PS19 mice.**

(**A, B**) Western blotting analysis and quantification of AT8, AT100, PHF13 and tau13 in the supernatant and pellet fraction of hippocampal extracts from WT and PS19 mice in the preventative cohort (7-month-old). n = 5 per group. (**C, D**) Western blotting analysis and quantification of AT8, AT100, PHF13 and tau13 in the supernatant and pellet fraction of cortex from WT and PS19 mice in the preventative cohort (9-month-old). n = 5 per group. Statistical significance was determined by unpaired Student’s t test. Data are shown as mean ± SEM, **p* < 0.05, ***p* < 0.01, ****p* < 0.001.

**Supplementary Figure 5**


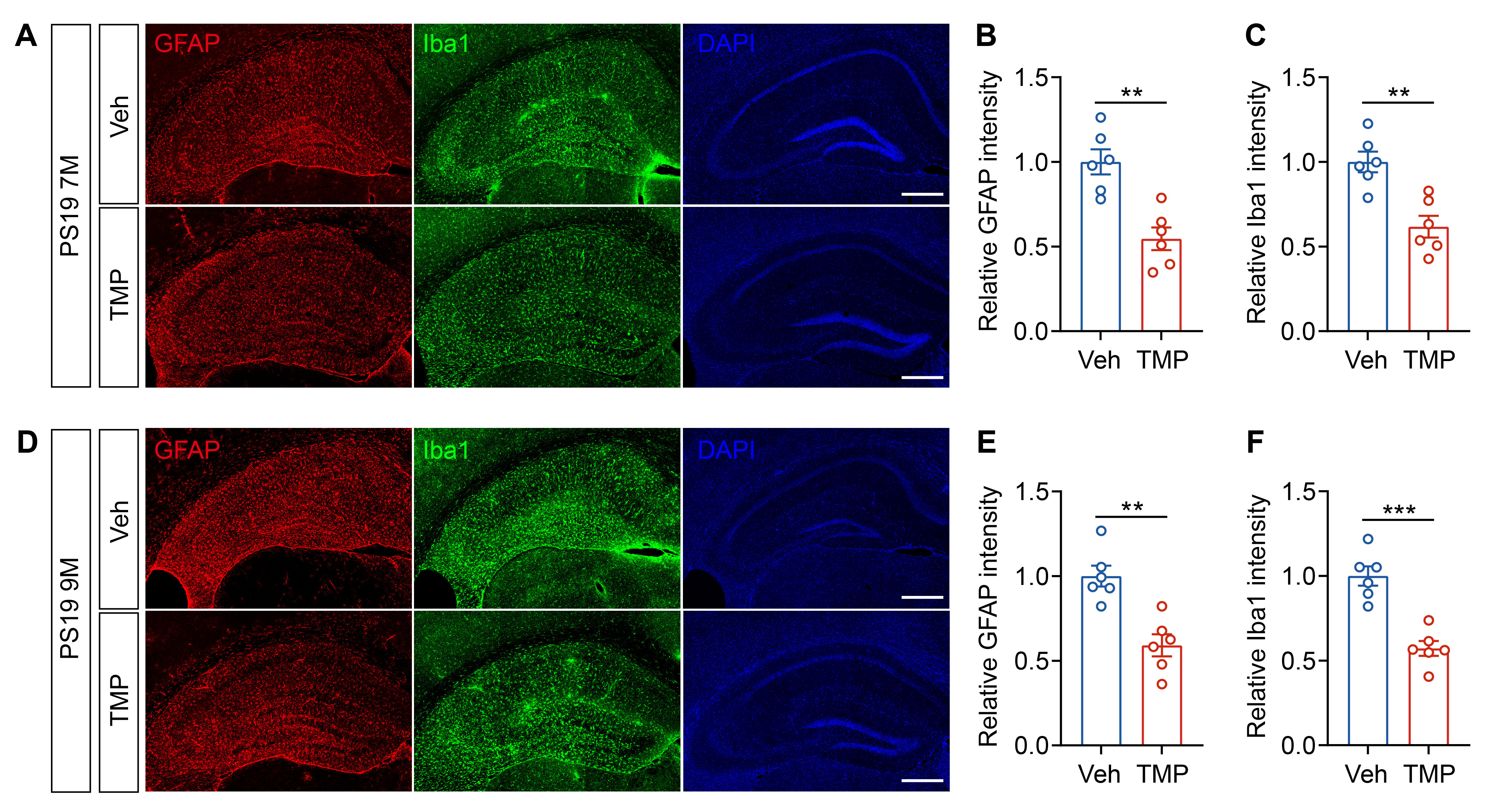


**Supplementary Figure 5 TMP195 treatment attenuates gliosis in young and aged PS19 mice.**

(**A**) Representative images of astrogliosis (GFAP) and microgliosis (Iba1) in the hippocampus of preventative cohort (7-month-old) PS19 mice administrated with TMP195 or vehicle. Scale bar: 200 μm. (**B**, **C**) Quantification of GFAP and Iba1 intensity in A normalized to control vehicle, n = 6 per group. (**D**) Representative images of astrogliosis (GFAP) and microgliosis (Iba1) in the hippocampus of treatment cohort (9-month-old) PS19 mice administrated with TMP195 or vehicle. Scale bar: 200 μm. (**E**-**F**) Quantification of GFAP and Iba1 intensity in D normalized to control vehicle. n = 6 per group. Statistical significance was determined by unpaired Student’s t test. Data are shown as mean ± SEM, ***p* < 0.01, ****p* < 0.001.

**Supplementary Figure 6**


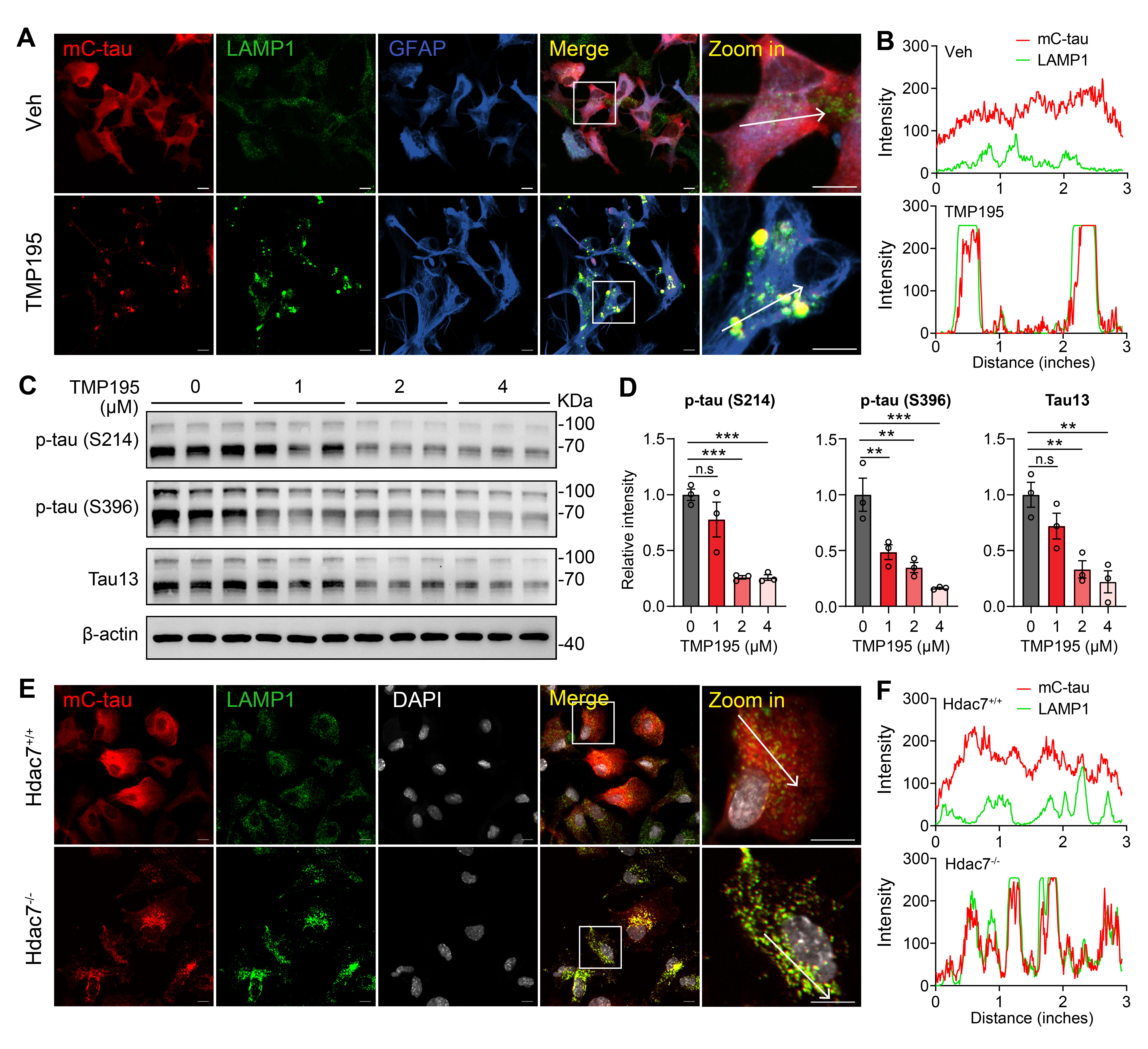


**Supplementary Figure 6 Inhibition or deletion of HDAC7 enhances lysosomal tau degradation in primary astrocytes.**

(**A**) Representative fluorescence images showing colocalization of mCherry-tau and lysosomes (LAMP1) in primary astrocytes (GFAP) infected with LV-G1-mCherry-tau (human P301S-tau, 1N4R) and then treated with TMP195 or vehicle for 24 h. (**B**) The fluorescence intensity profile along the lines indicated in A. (**C, D**) Western blotting analysis and quantification of p-tau (S214), p-tau (S396) and tau13 in P301S tau-overexpressing astrocytes treated with TMP195 for 24 h. n = 3 per group. (**E**) Representative fluorescence images showing colocalization of mCherry-tau and lysosomes (LAMP1) in control and HDAC7 knockout astrocytes infected with LV-G1-mCherry-tau. (**F**) The fluorescence intensity profile along the lines indicated in E. Data are shown as mean ± SEM, ***p* < 0.01, ****p* < 0.001, n. s, not significant.

**Supplementary Figure 7**


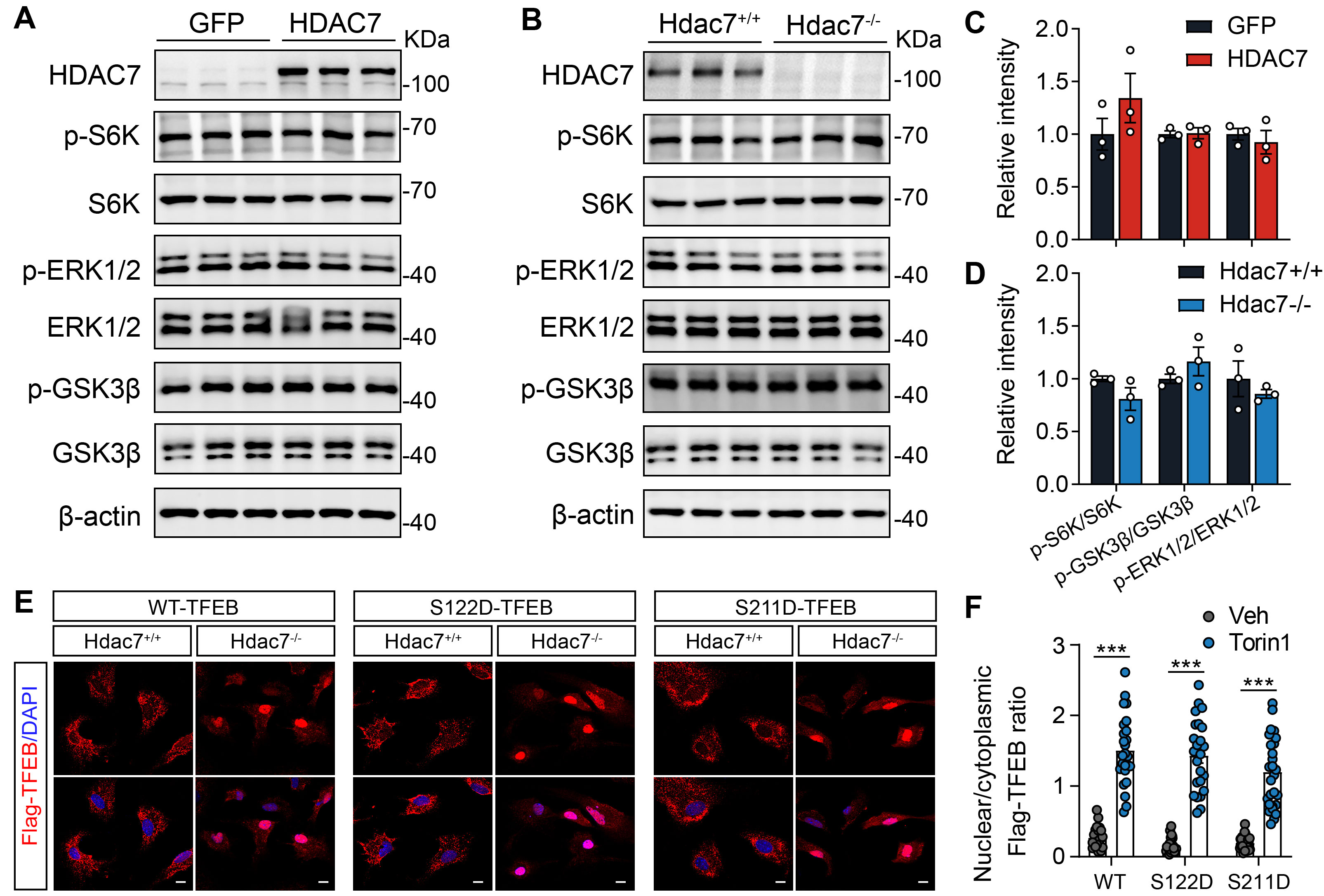


**Supplementary Figure 7 Overexpression or deletion of HDAC7 has no effects on the activity of TFEB upstream kinases.**

(**A-D**) Western blotting analysis and quantification of HDAC7, p-S6K, S6K, p-ERK1/2, ERK1/2, p-GSK3β and GSK3β in HDAC7-overexpressing or HDAC7 knockout primary astrocytes. n = 3 per group. (**E**) Representative immunostaining images of WT/S122D/S211D Flag-TFEB in control and Torin1-treated astrocytes. Scale bar: 10 μm. (**F**) Quantification of Flag-TFEB nuclear/cytoplasmic ratio in E. n = 24 to 28 cells per group. Statistical significance was determined by unpaired Student’s t test. Data are expressed as mean ± SEM, ****p* < 0.001.

**Supplementary Figure 8**


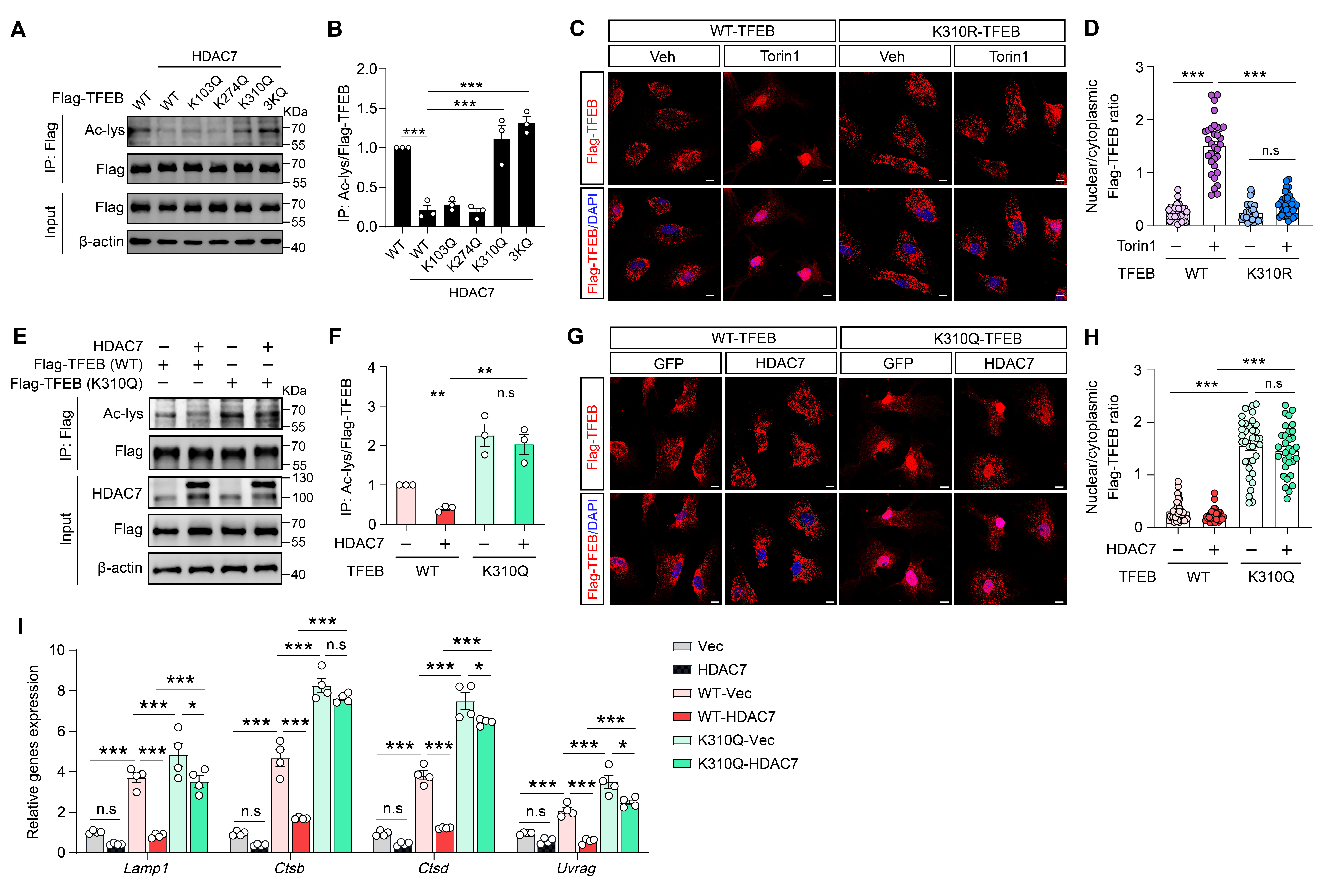


**Supplementary Fig. 8 Acetyl-mimic mutation of TFEB at K310 rescues HDAC7 overexpression-induced impairments of TFEB-lysosome signaling.**

(**A, B**) Immunoprecipitation assay showing the acetylation level of exogeneous WT or mutated (K to Q) Flag-TFEB in control and HDAC7-overexpressing HEK293T cells. (**C**) Representative immunostaining images of WT/K310R Flag-TFEB in control and Torin1-treated astrocytes. Scale bar: 10 μm. (**D**) Quantification of Flag-TFEB nuclear/cytoplasmic ratio in C. n = 31 (Veh+WT-TFEB), 32 (Torin1+WT-TFEB), 30 (Veh+K310R-TFEB), 35 (Torin1+K310R-TFEB). (**E, F**) Immunoprecipitation assay showing the acetylation level of exogeneous WT or K310Q mutated Flag-TFEB in control and HDAC7-overexpressing primary astrocytes. The acetylation level of Flag-TFEB was quantified. n = 3 per group. (**G**) Representative immunostaining images of WT/K310Q Flag-TFEB in control and HDAC7-overexpressing astrocytes. Scale bar: 10 μm. (**H**) Quantification of Flag-TFEB nuclear/cytoplasmic ratio in C. n = 34 (Vec+WT-TFEB), 30 (HDAC7+WT-TFEB), 35 (Vec+K310Q-TFEB), 32 (HDAC7+K310Q-TFEB). (**I**) Analysis of Lamp1, Ctsb, Ctsd and Uvrag mRNA levels by RT-qPCR in primary astrocytes overexpressed with HDAC7 and WT/K310Q Flag-TFEB. n = 4 per group. Statistical significance was determined by one-way ANOVA with Tukey’s post hoc analysis. Data are shown as mean ± SEM, **p* < 0.05, ***p* < 0.01, ****p* < 0.001, n. s, not significant.

**Supplementary Figure 9**


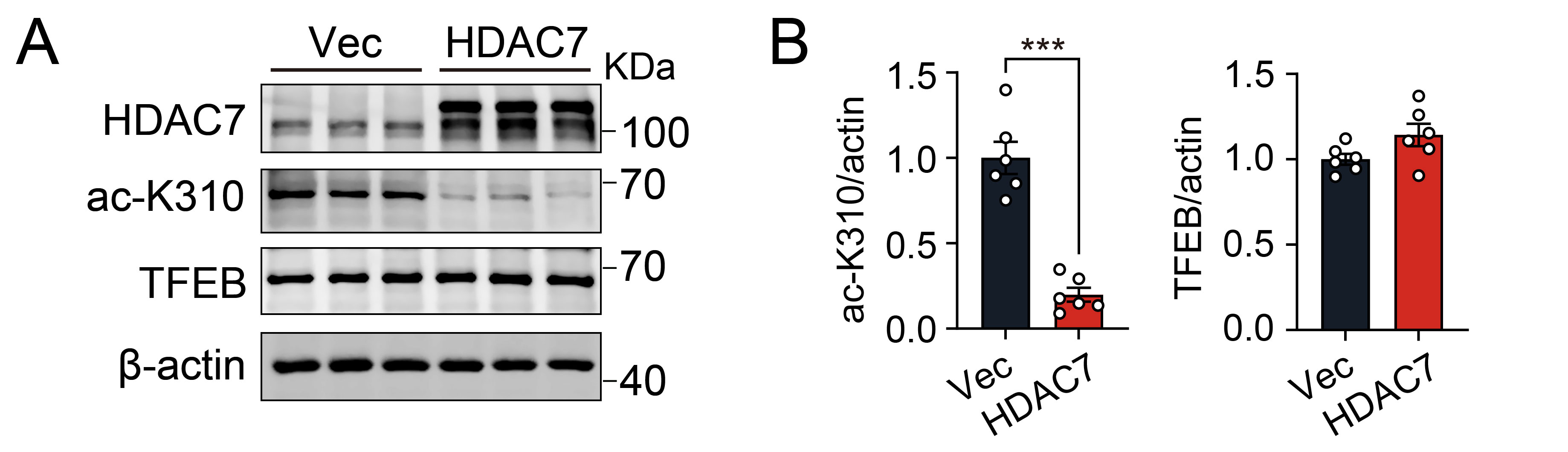


**Supplementary Figure 9 Overexpression of HDAC7 inhibits TFEB acetylation at K310.**(**A, B**) Western blotting analysis and quantification of ac-K310 and total TFEB in GFP and HDAC7-overexpressing astrocytes. n = 6 per group. Statistical significance was determined by unpaired Student’s t test. Data are shown as mean ± SEM, ****p* < 0.001.

**Supplementary table 1 Primary antibodies used in the study**

| Antibodies | Applications | Source | Catelogy |
| --- | --- | --- | --- |
| Rabbit monoclonal recombinant anti-HDAC4 antibody [EPR22937-157] | WB: 1:500 | Abcam | Cat# ab12171 |
| Rabbit polyclonal anti-HDAC5 antibody | WB: 1:500 | Abcam | Cat# ab55403 |
| Rabbit polyclonal anti-HDAC7 antibody (KG-17) | WB: 1:500 | Sigma-Aldrich | Cat# H2662 |
| Rabbit polyclonal anti-HDAC9 antibody | WB: 1:500 | Sigma-Aldrich | Cat# SAB4503694 |
| Mouse monoclonal anti-phospho-tau (Ser202, Thr205) antibody (AT8) | WB: 1:500  IF: 1:200  IHC: 1:250 | ThermoFisher | Cat# MN-1020 |
| Mouse monoclonal anti-phospho-tau (Thr212, Ser214) antibody (AT100) | WB: 1:1000 | ThermoFisher | Cat# MN-1060 |
| Mouse monoclonal anti-phospho-tau (Ser396) antibody (PHF13.6) | WB: 1:1000 | ThermoFisher | Cat# 35-5300 |
| Rabbit polyclonal anti-acetyl-tau (K174) antibody | WB: 1:1000 | Signalway Antibody | Cat# HW181 |
| Mouse monoclonal anti-tau antibody [tau-13] | WB: 1:1000 | Biolegend | Cat# 835204 |
| Chicken polyclonal anti-GFAP antibody | IF: 1:500 | Abcam | Cat# ab4674 |
| Mouse monoclonal anti-GFAP antibody (GA5) | IF: 1:500 | Cell signaling | Cat# 3670 |
| Rabbit polyclonal anti-Iba1 antibody | IF: 1:500 | WAKO | Cat# 019-19741 |
| Rabbit monoclonal anti-NeuN antibody [EPR12763] | IF: 1:500 | Abcam | Cat# ab177487 |
| Rabbit polyclonal anti-LAMP1 antibody | WB: 1:1000  IF: 1:150 | Abcam | Cat# ab24170 |
| Mouse monoclonal anti-LAMP1 antibody (D4O1S) | IP: 1:80 | Cell signaling | Cat# 15665 |
| Rabbit monoclonal anti-DYKDDDDK (Flag) antibody (D6W5B) | WB: 1:1000  IP: 1:200  IF: 1:500 | Cell signaling | Cat# 14793 |
| Rabbit polyclonal anti-Acetylated-Lysine Antibody | WB: 1:1000 | Cell signaling | Cat# 9441 |
| Rabbit polyclonal anti-TFEB antibody | WB: 1:1000  IP: 1:100  IF: 1:100 | Proteintech | Cat# 13372-1-AP |
| Rabbit polyclonal anti-ac-TFEB (K310) antibody | WB: 1:500  IF: 1:100 | Ye Lab | N/A |
| Rabbit recombinant monoclonal anti-Cathepsin B antibody [EPR21033] | WB: 1:1000 | Abcam | Cat# ab214428 |
| Rabbit recombinant monoclonal anti-Cathepsin D antibody [EPR3057Y] | WB: 1:1000 | Abcam | Cat# ab75852 |
| Mouse monoclonal anti-β-Actin antibody (13E5) | WB: 1:1000 | Cell signaling | Cat# 4970 |
| Rabbit recombinant monoclonal anti-p70 S6 kinase antibody (E8K6T) | WB: 1:1000 | Cell signaling | Cat# 34475 |
| Rabbit recombinant monoclonal anti-phospho-p70 S6 kinase (Thr389) antibody (108D2) | WB: 1:1000 | Cell signaling | Cat# 9234 |
| Rabbit monoclonal anti-GSK3 beta antibody [Y174] | WB: 1:1000 | Abcam | Cat# ab32391 |
| Rabbit monoclonal anti- phospho-GSK3 beta (Ser9) antibody [EPR2286Y] | WB: 1:1000 | Abcam | Cat# ab75814 |
| Rabbit recombinant monoclonal anti-p44/42 MAPK (Erk1/2) antibody (137F5) | WB: 1:1000 | Cell signaling | Cat# 4695 |
| Rabbit recombinant monoclonal anti-phospho-p44/42 MAPK (Erk1/2) (Thr202/Tyr204) antibody (D13.14.4E) | WB: 1:1000 | Cell signaling | Cat# 4370 |

**Supplementary table 2 Information for human brain samples**

| Case number | Gender | Age | Neuropathological Diagnosis | Postmortem Interval (h) |
| --- | --- | --- | --- | --- |
| PTB078 | Female | 86 | AD | 6.33 |
| PTB079 | Female | 80 | AD | 18 |
| PTB083 | Female | 100 | AD | 3 |
| PTB108 | Male | 72 | AD | / |
| PTB114 | Female | 80 | AD | 13 |
| PTB129 | Male | 83 | AD | 4.5 |
| PTB139 | Male | 82 | AD | 38 |
| PTB041 | Male | 80 | Control | 7.5 |
| PTB054 | Female | 84 | Control | 20 |
| PTB088 | Male | 79 | Control | 29 |
| PTB140 | Male | 83 | Control | 38 |
| PTB144 | Female | 96 | Control | 32 |
| PTB158 | Male | 86 | Control | 5 |
| PTB186 | Female | 87 | Control | 12.5 |

**Supplementary table 3 Primers used for RT-qPCR**

| Genes | Forward (5’ to 3’) | Reverse (5’ to 3’) |
| --- | --- | --- |
| Hdac4 | AGCAGCACCAGCAGTTCCT | CCGCTTCTTCCTCCTCACTCT |
| Hdac5 | AGCAGCACCAGCAGTTCCT | CACTCTCGCCATCCTCATCCT |
| Hdac7 | CGCAGCCAGTGTGAGTGTCT | GCTCGTTCCAGATGGTGTCAGTA |
| Hdac9 | GATGATGATGCCTGTGGTGGAT | TGCTGCTGCTGCTGAATAAGAA |
| Lamp1 | GCCTCTATGGCACTGCAACT | TTTGGGCTGATGTTGAACGC |
| Ctsb | TGACCGAACCTGCATTCACA | ATGCTCCAGAGGGATAGCCA |
| Ctsd | CCACGGAGCCAGTGTCAGAGTTA | CCACAGGTTAGAGGAGCCAGTATCA |
| Ctsf | GAGCCACCTTGCAATGATCC | TCACAGAGAAGTCCTGGGGC |
| Map1lc3b | GCCTTCTTCCTCCTGGTGAA | TGCTGTCCCGAATGTCTCC |
| Atg9b | TCATCAACAGCAGCAGCAAGAACT | AAGCAGGACTGGAGCCATCACT |
| β-actin | CCACCATGTACCCAGGCATT | CGGACTCATCGTACTCCTGC |
